# Electrode Nanostructures in Lithium‐Based Batteries

**DOI:** 10.1002/advs.201400012

**Published:** 2014-12-29

**Authors:** Nasir Mahmood, Yanglong Hou

**Affiliations:** ^1^ Department of Materials Science and EngineeringCollege of Engineering, Peking UniversityBeijing100871China

**Keywords:** lithium ion battery, lithium air battery, lithium sulfur battery, electrode nanostructures, lithium metal anode

## Abstract

Lithium‐based batteries possessing energy densities much higher than those of the conventional batteries belong to the most promising class of future energy devices. However, there are some fundamental issues related to their electrodes which are big roadblocks in their applications to electric vehicles (EVs). Nanochemistry has advantageous roles to overcome these problems by defining new nanostructures of electrode materials. This review article will highlight the challenges associated with these chemistries both to bring high performance and longevity upon considering the working principles of the various types of lithium‐based (Li‐ion, Li‐air and Li‐S) batteries. Further, the review discusses the advantages and challenges of nanomaterials in nanostructured electrodes of lithium‐based batteries, concerns with lithium metal anode and the recent advancement in electrode nanostructures.

## 1 Introduction

The huge consumption of fossil fuels by the increasing population is ringing the alarm bell for the environment. Thus, green energy has attracted intensive attentions since current “fossil fuels” are being depleted with the developing society and concerns about environment have become serious.^1^ To resolve these problems and to decrease the usage of fossil fuels, alternative renewable energy production technologies need to be utilized such as fuel cell and solar cell, hydro‐power and wind‐energy.^2^ These energy production systems need storage devices, thus it is highly desirable to develop efficient and cheaper energy storage devices to meet the requirement of advanced society.^3^, ^4^, ^5^, ^6^ Among various energy storage systems, rechargeable batteries are one of the most suitable and feasible options for the storage of electrical energy.^7^, ^8^, ^9^, ^10^ Several rechargeable battery systems like lead‐acid, nickel‐metal hydride, nickel‐cadmium and lithium‐based batteries are serving mankind from last one century. However, advancement in portable electronics and their high demands need faster and safer energy storage systems.^5^, ^11^, ^12^, ^13^, ^14^, ^15^, ^16^, ^17^, ^18^, ^19^ Furthermore, the dream of electrification of the road market and electricity storage from intermittent energy production systems needs advanced rechargeable batteries.^20^ Thus, several key parameters, e.g., cost, high energy and power, longer cyclic life, safety and environmental benignity, are needed to be considered in the development of advance rechargeable batteries to make them useful in hybrid electric vehicles (HEVs) and electric vehicles (EVs).^21^ It is noted that the low specific energy or energy density limit the most mass producible EVs to a short traveling distance (≈163 km) per charge.^22^ Furthermore, the complex systems result in high cost and make batteries thermodynamically unsafe. In this regard, the United States Advanced Battery Consortium (USABC) has introduced several standards and put forward long‐term objectives for batteries of EVs to lead the researchers in this field.^23^ The required specific energy and energy density values determined by USABC for rechargeable battery systems of EVs are 200 Wh kg^–1^ and 300 Wh L^–1^ at a discharge rate of C/3 (C/3 means a complete discharge in 3 hours).^23^ Furthermore, the battery should be able to cycle 1000 times with at least 80% capacity retention.^24^ From the recent development, it is considered that lithium‐based batteries (lithium ion battery, lithium sulfur battery, and lithium air battery) are capable of achieving these standard values and making the longer drives of EVs possible.^25^, ^26^, ^27^, ^28^, ^29^ Unfortunately, several problems are a big hurdle in the real application of lithium‐based batteries in EVs, e.g., poor cyclic life and power density.^5^, ^30^ The development of new high capacity electrode materials that possess long cyclic life than traditional materials is a key solution to above mentioned problems.^31^ The high capacity materials have ability to increase the energy contents per volume and weight at low cost. But these materials introduced new fundamental challenges both at their synthesis and operation as an electrode in batteries.^12^, ^32^, ^33^ Progress in lithium‐based batteries has been largely benefited by developing nanostructured electrodes in comparison to conventional electrode. The high active surface area of nanostructures significantly improves the efficiency to completely utilize the electrode material, resulting in enhanced performance of electrode.^34^, ^35^, ^36^ The conventional lithium ion (Li‐ion) batteries are made of graphite which acts as negative electrode and metal oxides or phosphates (LiCoO_2_, LiFePO_4_, etc.) as positive electrode.^37^ All these materials working as anode and cathode store lithium ions (Li^+^) through insertion process, thus resulting in limited energy storage due to limited available vacancies for Li^+^ in the host sites.^38^ However, the insertion mechanism brings good capacity retention because Li^+^ only resides at host sites and can be cycled easily without destroying the host structure. In contrast, to improve the specific capacity of Li‐ion battery new anode materials are introduced which store Li^+^ in different way.^39^, ^40^ These negative electrode materials (e.g., Si, Ge or Sn etc.) utilized the alloying or conversion reaction with Li^+^ by breaking the bonds between the host atoms, thus enhanced the capacitive performance of electrode.^13^, ^41^, ^42^, ^43^ In terms of theoretical capacity the Si‐Li, Ge‐Li and Sn‐Li alloy brings capacities as high as 4200, 1623, 994 mAh g^–1^, respectively but result in volume expansion up to 300%.^44^, ^45^, ^46^, ^47^, ^48^ Such drastic structural changes happen as result of bond breaking of host atoms during alloy formation that affect the capacity retention and cyclic life drastically.^49^, ^50^ The capacity decay could also arises due to electrical insulation of the fractured electrode material.^51^, ^52^ Thus a critical design at nanoscale and good control on structure is required to overcome volume changes, structural fracture and side reactions.

To overcome the challenges and limitations of alloy forming negative electrodes of Li‐ion battery, new energy storage mechanisms are introduced utilizing the similar concept with Li‐ion battery to improve its energy density equivalent to that of gasoline.^53^ This concept based on the conversion reaction of Li^+^ with oxygen and sulfur give rise to new types of lithium‐based devices named as lithium air (Li‐air) and lithium sulfur (Li‐S) batteries.^54^, ^55^, ^56^, ^57^, ^58^ High theoretical energy densities of 3445 and 2600 Wh kg^–1^ for Li‐air and Li‐S batteries can be achieved considering a complete conversion reaction of Li^+^ with oxygen and sulfur to form Li_2_O_2_ and Li_2_S, respectively.^59^, ^60^, ^61^ At fully charged state excluding oxygen the specific energy density of Li‐air battery (11 680 Wh kg^–1^) reaches to the energy density of the gasoline (13 000 Wh kg^–1^).^62^ However, several challenges from cell assembly to working materials including electrolyte, lithium metal anode and cathode catalysts need to be addressed before its practical application.^63^, ^64^, ^65^ The delivery of pure air (O_2_) is a great challenge associated with Li‐air battery since air is a mixture of various gases and these gases act as poison for Li‐air cell and destroy its capacity resulting in poor cyclic life.^59^ Similarly, Li‐S battery has its own limitation at cell operation associated with large volume changes (80%), poor conductivity and polysulfide anions shuttle that destroyed the internal operation system of the cell and affect its performance.^60^ Thus, development of sulfur nanostructures or its hybrids with other metals or/and carbon are necessary to overcome these issues.^66^, ^67^ Please note that the present review only discusses critical challenges associated with electrode of lithium‐based batteries and their proposed solution with recent examples from literature, thus we apologize to the authors if their work is not deliberated below.

This review summarizes the recent developments in lithium‐based batteries, different chemistries of lithium‐based batteries and electrode nanostructures, challenges associated with these nanostructures and their solutions. It includes the contributions both from the author's group and scientific community to highlight the advancement and important aspects on lithium‐based batteries. It will first explain the working principals of lithium‐based batteries (Li‐ion, Li‐air, and Li‐S). It will also outline the various challenges associated with lithium‐based batteries both at electrode structure and cell operation. Moreover, it will present the advantages and disadvantages of nanostructures over the conventional anode and cathode materials. Further, it will summarize the various methodologies developed to overcome the challenges associated with electrode nanostructures both at the level of synthesis and structure design. The controlled synthesis leading to advanced electrode materials and catalysts, providing an ideal system to study synergistic effect of various hybrid structures for enhanced performance. The review will lead towards the future of lithium‐based systems (Li‐ion, Li‐air and Li‐S) with possible solution to the associated problems.

## 2 Working Principle and Structure of Lithium‐Based Batteries

The working mechanism and structure of each type of cell is elaborated in the respective sections. In general all types of batteries (Li‐ion, Li‐air and Li‐S) have similar cell assembly that consists of two electrodes (anode and cathode/catalyst) and electrically insulating separator but are permeable for ions and electrolyte. However, keep in mind that Li‐air cell also needs a continuous oxygen supply. But the cell chemistries in all these lithium‐based systems are quite different from each other; this is explained below.

### 2.1 Li‐Ion Battery

**Figure 1:**
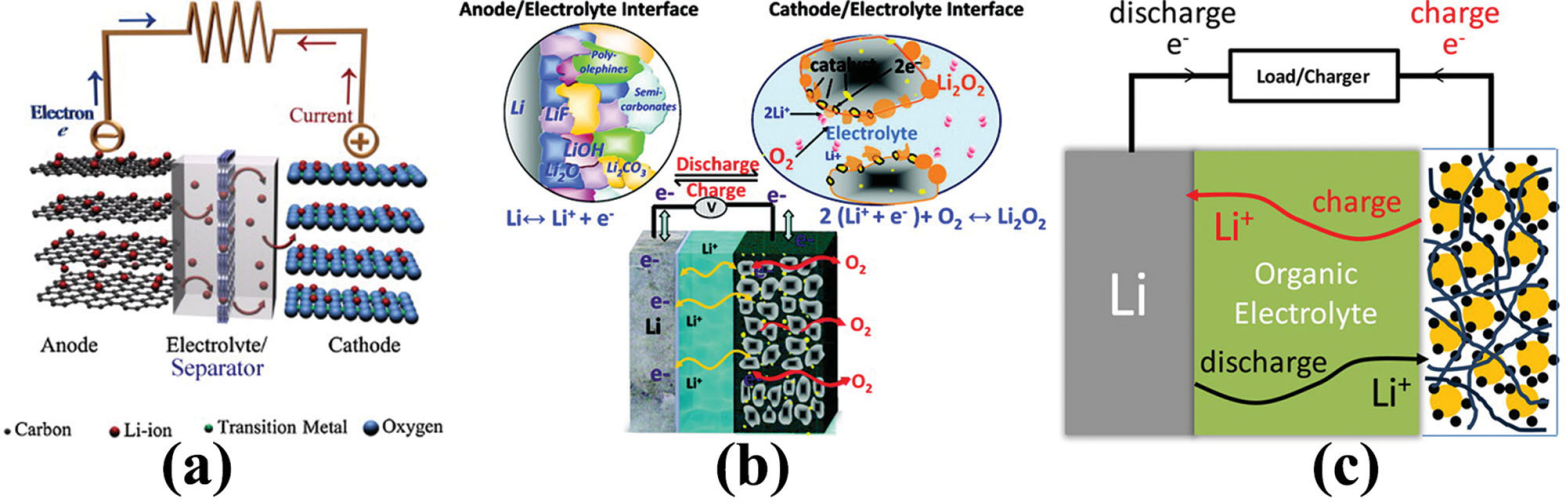
Schematic representation of structure and working of a) Li‐ion battery, b) Li‐air battery c) Li‐S battery. Part (a) reproduced with permission.^53^ Copyright 2011, Elsevier. Part (b) reproduced with permission.^28^ Copyright 2014, American Chemical Society. Part (c) reproduced with permission.^150^ Copyright 2010, American Chemical Society.

The Li‐ion battery received tremendous attention of researchers and became the major source of energy storage in portable electronics after the first release by the Sony company in early 1990s.^68^ The fundamental structure of Li‐ion battery consists of two electrodes (the anode acts as the negative electrode and the cathode acts as the positive) and electrolyte capable of ionic conduction but act as insulator for electrons. A permeable polymeric separator that allows free movement of ions but inhibits the conduction of electrons to prevent the short circuiting of the battery. In general, during the discharge process, Li^+^ moves spontaneously from the negative electrode towards the positive one through the electrolyte. Consequently, an electron flows from the negative to the positive electrode via an external circuit generating an electrical power, as shown in **Figure** **1**a. A reverse process occurs during charging for both Li^+^ and electrons as they move from the positive to the negative electrode. The chemical reactions occurring both at the anode and the cathode of a commercial Li‐ion battery is represented below.

(1) $${\mathrm{LiCoO}}_{2}↔{\mathrm{Li}}_{1-x}{\mathrm{CoO}}_{2}+{\mathrm{xLi}}^{+}+{\mathrm{xe}}^{-}$$

(2) $$6C+{\mathrm{xLi}}^{+}+{\mathrm{xe}}^{-}↔{\mathrm{LiC}}_{6}$$

If *x* is equal to 0.5 then the half reaction at the negative electrode will deliver its theoretical capacity of 372 mAh g^–1^. Furthermore, in the case of overcharge up to 5.2 V and over‐discharge or supersaturating the cathode, this results in the production of irreversible byproducts leading to destruction of cell. The respective chemical reactions are presented in Equations (3) and (4), respectively.^69^

(3) $${\mathrm{LiCoO}}_{2}\to {\mathrm{CoO}}_{2}+e^{-}+{\mathrm{Li}}^{+}$$

(4) $${\mathrm{LiCoO}}_{2}+e^{-}+{\mathrm{Li}}^{+}\to {\mathrm{Li}}_{2}O+\mathrm{CoO}$$

### 2.2 Li‐Air Battery

The Li‐air battery consists of a similar assembly to Li‐ion battery: an anode of lithium metal or lithiated compounds of other metals, an electrolyte, a separator and a “breathing” air electrode that has the ability to catalyze O_2_. The general cell assembly for Li‐air battery is presented in Figure 1b. On the basis of electrolyte, the Li‐air battery is divided into four categories: aqueous, non‐aqueous, hybrid, and all solid‐state battery.^53^ But with the introduction of non‐aqueous chemistry late in the 1990s, it has received tremendous attention from researchers over aqueous chemistry because of its higher theoretical specific energy.^59^ The subjected reversible reaction for a non‐aqueous (aprotic) Li‐air cell is provided in Equation (5),(6).^53^ Initially, in the discharge process, Li^+^ is released from the anode and reacts with dissolved oxygen in the electrolyte on the surface of the porous cathode catalyst.^71^ The discharge product (Li_2_O_2_) has very low solubility and deposits on the pores of cathode catalyst.^72^ In reverse, during the charging process, oxygen gas is evolved from a catalyst electrode and Li metal is plated back onto the surface of the anode.^62^, ^73^, ^74^

(5) $$\mathrm{Li}↔{\mathrm{Li}}^{+}+e^{-}$$

(6) $$2{\mathrm{Li}}^{+}+2e^{-}+O_{2}↔{\mathrm{Li}}_{2}O_{2}$$

### 2.3 Li‐S Battery

A Li‐S battery is composed of four main components similar to other lithium‐based batteries: an anode composed of lithium metal/lithiated compound of other metals, an organic electrolyte, a separator and a cathode consisting of sulfur or hybrids of sulfur with other metals/carbon. Cell operation starts with the discharge reaction due to the charged state of sulfur cathode. Initially at the anode, the oxidation reaction occurs to release Li^+^ and electrons in the system. Thus, as the produced Li^+^ and electrons move towards the positive electrode through the electrolyte and external circuit, this respectively results in an electrical current in the circuit. Consequently, Li^+^ and electrons reduce the sulfur at the cathode surface to produce the lithium sulfide. The chemistry of the overall cell is explained by the chemical reactions presented in Equation (7), (8), and (9).^17^, ^28^, ^60^, ^75^

(7) $$\mathrm{Li}↔{\mathrm{Li}}^{+}+e^{-}$$

(8) $$2{\mathrm{Li}}^{+}+2e^{-}+S↔{\mathrm{Li}}_{2}S$$

Overall reaction of the complete cell

(9) $$2\mathrm{Li}+S↔{\mathrm{Li}}_{2}S$$

Sulfur atoms show strong tendency to catenation and form various homo‐atomic chains or cyclic rings. Among them orthorhombic *α*‐S_8_ is most stable at room temperature and when react with Li^+^ cyclo‐S_8_ reduces and form higher order polysulfide anions. Further with continuous reaction lower order polysulfide are also formed which are soluble in organic electrolytes.^76^, ^77^, ^78^

## 3 Scientific Challenges Associated with Lithium‐Based Batteries

Future research demands nanostrucutured materials with high active surface areas which could offer large sites for intercalation of Li^+^ for both insertion and conversion processes ultimately providing oppurtunities to tailor high energy density/power density devices for practical applications. However, a great advancement in the field of lithium‐based batteries has overcome many of the challenges that are big obstacles in their application to the field of portable electronics or short distance traveling EVs. Despite these considerable advancements, there still remains a number of challenges to be addressed before the usage in long distance traveling or heavy duty storage systems, e.g., storage of electricity in grid station or from the intermittent energy productions systems such as solar cells.

### 3.1 Challenges associated with Li‐Ion Batteries

The major challenges associated with electrode materials of Li‐ion battery are listed here with thier possible solutions. Due to these problems, the obtainable capacity is lower than the theoretical capacity and results in lower energy density that is insufficient for the intended applications. A low energy efficiency could arise due to large polarization, which becomes more severe at higher charge–discharge rates along wtih poor cyclic life due to capacity fading with successive cycles.

#### 3.1.1 Low Conductivity and Thermal Runway

It is a well‐known fact that most of the electrode materials bear low conductivities that cause poor conduction of electrons, which affect the storage of lithium. In addition, it is necessary that every material should have sufficient ability to efficiently receive the electrons and to deliver to the external circuit. Furthermore, due to low intrinsic conductivities, several materials have failed to utilize all of their redox sites, as no pathways exist for electron transfer between redox site and external circuit.^79^ Incorporation of carbon or other highly conductive agent as outer coatings or part of inner structure will effectively overcome this problem.^80^ Furthermore, the power of the battery depends strongly on internal impedance (impedance inversely interacts with power) and heat generated during operation. The heat generation inside the Li‐ion battery is due to three main factors: interfacial kinetics (activation), species transport (concentration), and heat production by the movement of charged products/electrons (ohmic) losses. The main heat generation happens due to the irreversibility in battery and from electrochemical reactions. The concentration gradient of species also affects the heat generation, but it is very small in well‐designed batteries.^81^ By developing appropriate cell chemistries and control over voltage range, thermal issues can be resolved.

#### 3.1.2 Morphological and Structural/Phase Changes

Lithium storage in several materials strongly depends on their morphology, thus if morphology of electrode material changes during lithium insertions and extraction, it could drastically affect the performance and cyclic life. Furthermore, any phase or morphology changes might cause the aggregation of newly segregated product resulting in loss of the electrical contact, ultimately leading to destruction of cell operation. Further the morphological changes also brings fresh surface of electrode materials in contact with electrolyte that rebuild solid electrolyte interface film (SEI) which cause irreversible loss of capacity.^33^, ^82^ Similar to morphological changes, several materials show crystal structure or phase changes during cycling process of electrode. The newly formed crystal structure or phase thus behaves differently towards lithium storage. Most crystal structure changes cause poor electronic conductivity, sluggish kinetics towards lithium insertion and extraction as a result of poor capacity and cyclic life.^83^ These structural changes are inherent to specific material at specified conditions. Nanoscience plays a critical role by developing nanostructures that strongly diminishes the structural changes by improving phase transformation kinetics.

#### 3.1.3 Volume Changes and Thickness of Solid Electrolyte Interface Film

The volume changes of electrode materials during alloying and de‐alloying with Li^+^ result in severe volume increase that produces strains in the electrodes. Thus, as a result, isolation of the electrode material from the current collector causes electrical insulation or short circuiting of cell. Further, because of electrical insulation, the inaccessibility of Li^+^ to access the redox sites in active materials cause drastic capacity fading and poor cyclic life. These volume changes are more associated with the high theoretical capacity materials such as Si, Ge, and Sn.^44^, ^84^, ^85^ Thus, reduction in size to nano, outer protection coatings, void engineering, self‐healing polymeric matrix and hard metal (Co, Ni, Fe, etc.) doping are possible ways to resolve these problems. Furthermore, the organic electrolytes decompose at the working potential of <0.5 vs. Li/Li^+^ and forms thin SEI film on the surface of electrode.^52^ Thus cause an irreversible loss of capacity, but it is one fundamental part of battery operation that cannot be completely diminished but a good control over thickness of SEI film can be achieved by tuning the surface chemistry. However, the problems lie in the poor mechanical strength of SEI film, as it cannot bear any stress and thus cannot withstand any pressure or stress. What happens during the structural, morphological, and volume changes in the electrode materials causes the breakdown of SEI film and exposure of the fresh electrode surface to the electrolyte, resulting in further decomposition of the electrolyte and fading of capacity.^85^ Furthermore, the SEI film is an insulator (electronically) in nature and its regeneration increases the diffusion path for Li^+^. To this day, there is no good control over the chemical composition, grain size, thickness, and spatial distribution of the SEI film. In this case, nanostructures cause a big problem due to their high surface area and energies, but controlling these factors or protecting the surface with inactive metals or carbon coatings may result in better control over the SEI film.

### 3.2 Challenges associated with Li‐Air Batteries

In addition to the challenges presented for Li‐ion, the performance of Li‐air battery is also limited (well below its theoretical capacity) because of some fundamental issues related to its working and operation: it has a much poorer cyclic life than the available Li‐ion battery. These issues are related to obstacles in the air flow, deposition of discharge products and over‐potential with ongoing charge‐discharge process. Further poisoning of cathode catalysts by the other gases present in air (e.g., CO_2_, N_2_, NH_3_, H_2_O, etc.) decrease their efficiency.

#### 3.2.1 Air Transport and Deposition of Discharge Products

In contrast to the Li‐ion battery, the electrode of the Li‐air battery should have sufficient porosity and high conductivity to maintain the continuous transport of oxygen, Li^+^, and electrons towards redox sites deep in the electrode. The porous materials with sufficient conductivity are capable of carrying out the above mentioned operation through their pores (transport of oxygen and Li^+^) and solid network (electrons). In this regard, microscale materials (carbon‐based materials) with large pores to maintain the flow of required species (oxygen, Li^+^ and electrons) and decorated with nanoscale redox active materials (having capability to efficiently reduce the oxygen) are the best options to improve the efficiency and cyclic life of Li‐air battery. However, the deposition of discharge products (Li_2_O_2_ or LiO_2_) on the electrode is another issue that affects the efficiency of cathode catalyst and fade the capacity of the Li‐air battery, resulting in limited cyclic life. Basically, these depositions block the air pathway and cover the redox sites, thus inhibiting further catalysis of oxygen. Furthermore, the deposited product makes a continuous layer which causes electric shut down of battery due to its electrical insulation properties; further, the resistance of Li_2_O_2_ increases with increase of the thickness of the deposited film and causes a drop in working voltage. It is observed that Li_2_O_2_ is a bulk insulator in nature with a bandgap (DFT: > 1.88 eV and GW: 4.91 eV) which emphasizes that small increase in Li_2_O_2_ thickness will largly affect the electronic conduction. The proposed solution to this problem is to develop bi‐functional catalysts that have the ability to undergo oxygen reduction (discharging) and, in the reverse process, evolution of oxygen (charging) by breaking the deposited products and making the air flow pathways clear for the next discharge process.^71^

#### 3.2.2 Poison by Unwanted Gases

To commercialize the Li‐air cell, it is compulsory that it can work using oxygen from atmospheric air and deliver the same specific energy as obtained in the lab utilizing the pure oxygen. But when it is tested in atmospheric air, it fails to deliver the same performance because of insufficient supply of oxygen and presence of unwanted gases. Thus, it requires a compressor for continuous supply of air and purifier to remove the contents of carbon dioxide and water. These unwanted gases react with discharge products and stop the reversible cycling of battery. However, to this day, most of the work has been carried out on the cathode catalyst and electrolytes of Li‐air battery. Few reports have also presented the replacement of lithium metal anode with lithiated compounds to stop severe volume changes and unwanted reactions on the surface of anode, but no progress regarding this issue is reported yet.^59^

#### 3.2.3 Specific Catalyst Loading and Electrolyte Decomposition

An appropriate loading and distribution of an active catalyst on the cathode surface to operate the charging–discharging process is another complication associated with the Li‐air battery. The mostly active catalyst was supported on carbon and then attached with electron collector metal mesh using binder (non‐conductive in nature) similar to that of Li‐ion battery. Thus, homogenous dispersion in proper ratios of these three components to maintain the electrical conductivity is critical as use of a binder decreases the conductivity. To resolve this problem, the catalyst should be grown on a three‐dimensional (3D) carbon network with uniform distribution for direct use, which can provide multiple advantages such as better attachment and distribution of catalyst and free of non‐conductive binder.^86^ The second issue is related with the stability of the electrloyte, in this regard various kinds of electrolytes have been developed, including aqueous and nonaqueous electrolytes. However, both of them bring the fundamental issues and advantages: for example, the presence of H_2_O molecules in aqueous electrolyte is helpful to dissolve the reaction products, and thus inhibits the air blokage and mass loss, but the presence of water drastically affects the stability of the lithium anode. Thus, a sacrificial membrane or development of seramic SEI film can protect the lithium anode. The other class is nonaqueous electrolytes, which are free of water and follow the reaction mechanism through the formation of peroxide that is reversible in storage, but these have limited anodic oxidative stability due to the existance of carbonates. Thus the development of catalyst or electrolyte additives is the possible solution to this problem.

#### 3.2.4 Over‐Potential and Lithium Metal Anode

The working electrode potential of the Li‐air battery is 2.6–2.7 V, but it decreases during discharging while increasing during the charging process. This change in potential is called over‐potential and badly affects the performance of Li‐air cell. The over‐potential arises due to the deposition of discharge products (due to electrical loss as deposited products are insulater) during discharging process and failure of catalyst to completely recover the redox sites during charging. Another reason for increasing over potenstioal is mass and ionic loss due to the blockage of transport pathway by deposited products.^87^ Thus, highly efficient catalyst for both ORR and OER required to control the problem of cell over‐potential during the charge–discharge process. However, another issue related with Li‐air cell is use of lithium metal as electrode that is highly unstable in the cell environment. These metal electrodes react vigorously with electrolyte and decompose irreversibly, resulting in the formation of dendrites accompanied by large volume changes, consequently leading to destruction of cell. In most cases, formation of lithium hydroxide occures due to lithium reaction with hydroxyal ions produced from the decomposition of the electrolyte that limits the cyclic life of Li‐air battery. To avoid this problem, alternative anode materials need to be designed, such as lithiated compounds of silicon.^88^

### 3.3 Challenges associated with Li‐S Batteries

The Li‐S battery has almost similar challenges as associated with Li‐ion battery because of same working phenomenon and assembly of cell. Most of the challenges are associated with sulfur electrode due to its high resistance (10^−30^ S cm^–1^), higher volume changes (80%) during its conversion reaction with lithium.^89^ Besides these problems as described above in Li‐ion battery section, Li‐S battery has another very serious problem described below, several reports proposed nice solutions (presented in respective section) but yet not sufficient for practical applications.

#### 3.3.1 Polysulfide Anions Shuttle

Because of its unique structure sulfur shows strong catenation tendencies, as it forms homoatoms chains and cyclic rings. Among these, *α*‐S_8_ is more stable allotropic form of sulfur at ambient conditions. But during the discharge process lithium reduces and opens the cyclic rings of *α*‐S_8_, resulting in the formation of larger polysulfide anions of lithium Li_2_S_*x*_ (6 < *x* ≤ 8). Further as the discharge continue, the larger polysulfide anions are reduced to smaller ones Li_2_S_*x*_ (2 < *x* ≤ 6). Mostly, two discharge plateaus are present in the discharge curve of Li‐S cell at 2.3 and 2.1 V regardless the type of liquid electrolytes that shows the conversions of *α*‐S_8_ to Li_2_S_4_ and Li_2_S_4_ to Li_2_S.^28^ These polysulfide anions are soluble in organic solvents thus starts moving across the separator against their concentration gradient. Such a movement of polysulfide anions called shuttle transport and badly affect the transport of lithium in electrolyte. Furthermore, deposition of these polysulfide anions on the surface of electrodes results in insulation, negatively affecting the flow of electrons and as barrier to Li^+^ diffusion. This kind of movement further increases the internal temperature of cell and causes the thermal explosions of Li‐S battery.^60^ Thus, to avoid all these problems it is highly desirable to develop such electrode nanomaterials that can overcome the production of polysulfide anions or inhibit there dissolution in electrolyte.

## 4 Advantages and Challenges Associated with Electrode Nanostructures

**Figure 2:**
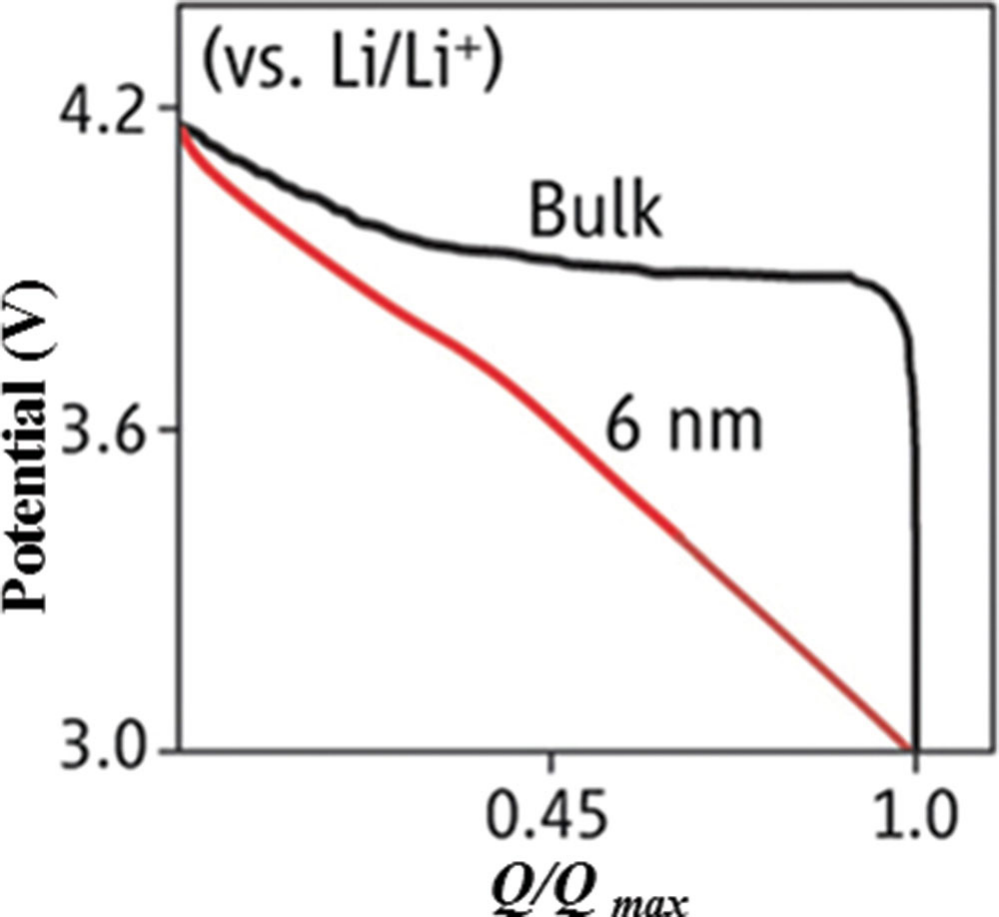
The discharge curves of nanocrystals and bulk material (LiCoO_2_). Reproduced with permission.^90^ Copyright 2014, American Association for the Advancement of Science.

It is well‐known fact that nanomaterials have a lot of advantages over their counter bulk materials, especially when we are talking about the electrode materials of lithium‐based batteries. **Figure** **2** represents the behavior of two electrodes composed of nanostructure and bulk; it is obvious that both structures behave differently and improved results are obtained for nanomaterials.^90^ Why nanomaterials behave differently is explained below, but a few disadvantages of nanostructures are also listed here, so readers should pay attention to them while developing their nanostructures for lithium batteries electrodes. These challenges are easily overcome by good control over structure and fabrication method or by developing hybrid structures.

### 4.1 Advantages of Nanostructures

#### 4.1.1 High Surface Area and Conductivities

Nanomaterials with a high surface‐to‐volume ratio increase the contact area between the electrolyte and electrode, resulting in more exposed redox sites which deliver higher power and energy density. Furthermore, availability of the larger surface area inhibits electrode polarization; in the case of the Li‐air battery, it offers more space for the redox reaction on surface, thus effectively improving the ORR and OER reaction and reducing electrode polarization.^91^ Furthermore, depositing the thin film of highly conductive metal or making a hybrid nanostructure with carbon the ionic and electronic conductivities of nanostructures boosts up and results in enhanced capacitance and cyclic life. This is because higher ionic and electronic conductivity provide a continuous flow of ions and electron to and from redox sites.^92^

#### 4.1.2 Mechanical Stiffness and Short Diffusion Path

The low‐dimension materials are mechanically strong and offer high resistance to any structural damage. Furthermore, their structure can easily be tuned to overcome the volume changes of the electrodes of lithium‐based batteries. Nano‐designing also inhibits any phase change of electrode material, thus preventing isolation of the electrode material and resulting in improved electrochemical properties.^93^ However, it is also obvious that nanomaterials have a smaller size in all dimensions, thus offering much shorter diffusion distances to ions and electrons and giving highly improved results. The diffusion distance is the most important factor in determining several characteristics of the battery, such as the potential variations, charge‐discharge time (power density) and ultimately capacitance. A higher diffusion path requires more time to reach the various redox sites and results in sluggish reaction kinetics.^85^

#### 4.1.3 New Lithium Storage Mechanism and Multiple Structural Designs

Nanomaterials offer new lithium storage mechanisms regardless of the simple accumulation of the ions at insertion sites, such as redox reactions at surface, storage of lithium at interfaces and nanopores which exist inherently in nanostructures. Furthermore, these storage mechanisms have no effect on the structure of electrode material, thus stable charge‐discharge can continue for a long time.^94^, ^95^ However, it is worth noting that single materials can be developed in various shapes (rods, plates, stars, particles, etc.) and dimensions (e.g., 0D, 1D, 2D, 3D). All of these different shapes and dimensions offer their own advantages and disadvantages for specific materials. The morphological designs that offer higher and easily available Li^+^ storage sites are better and can be selected according to nature of particular materials.^12^, ^96^ Nanostructures also offer hierarchical structures that can increase the oxygen flow in Li‐air battery and further offer the development of sponge‐like structures that are favorable to absorb the polysulfide anions in Li‐S batteries.

#### 4.1.4 Inactive‐Shift‐Active at Nano and Control Over Working Potential

It is observed that several materials which are inactive in their bulk states towards lithium storage can efficiently store the lithium at nanoscale and can perform the catalysis of oxygen. The reason behind such a shift in the nature of materials at nanoscale is because of the presence of several partially bonded atoms at surface and higher surface energies.^93^ Furthermore, Figure 2 clearly shows that by moving from bulk to nanoscale a clear change in the charge‐discharge curve is observed, which shows that nanomaterials completely act in new way than their bulk counterparts. As bulk materials show strong potential plateaus, they thus require higher current density, longer diffusion time, and different working potential for lithium storage, in contrast to nanomaterials that offer a shorter diffusion path.^90^

### 4.2 Challenges associated with Nanostructures

As well as various advantages of nanostructures, there are several challenges that need to be kept in mind before establishing a nanostructure for lithium‐based batteries.

#### 4.2.1 Higher Inter‐Particle Resistance and Low Tap Density

Nanomaterials show higher inter‐particle resistance and decrease the electrical connection among the particles. This resistance further increases if some volume changes occur in the electrode during reversible storage of lithium.^32^ It can be easily controlled by developing core‐shell structures or developing nanoclusters in which each nanocluster acts as single particle. Moreover, it is well‐known that nanomaterials have low tap density, thus offering low volumetric capacity. Higher thickness of electrode comes up at high mass loading that leads towards poor electrical and ionic conductivity during cycling. Finally, a small change at particle level can easily alter the electrical contact among the nanoparticles thus severely reduce the cycle life.^52^

#### 4.2.2 Agglomeration and Side Reaction

The smaller dimensions, higher surface energies, larger surface areas, and unsatisfied bonds of surface atoms result in severe agglomeration of nanomaterials that badly affects the lithium storage mechanism.^95^, ^96^ Further, because of higher surface energies and surface areas, nanomaterials are also involved in undesirable side reactions. However, if the structure is stable then with the formation of SEI film, the chances of side reactions are diminished.^85^ In addition, surface protection coatings or use of surfactants are other possible ways to inhibit the aggelomeration or side reactions.

#### 4.2.3 Thermodynamically Unstable and High Cost

Several nanomaterials are thermodynamically unstable under electrical fields, thus critically affecting their use as electrode in lithium batteries.^26^, ^97^ Furthermore, high cost and sophisticated synthesis methods are other big challenges in the real application of nanomaterials as electrodes of lithium‐based batteries. Thus, the development of cheap and industrially acceptable wet chemical routes is highly desirable for the real usage of nanomaterials.^97^

## 5 Advanced Electrode Nanostructures for Lithium‐based Batteries

Nanotechnology has a revolutionary role in determining the new electrode materials and their unique nanostructures to bring breakthroughs in the lithium‐based batteries towards their real applications in EVs. The following sections highlight the recent advancements in the electrode field of lithium‐based batteries including Li‐ion, Li‐air, and Li‐S batteries.

### 5.1 Architecture Anode Nanostructures for Li‐ion Batteries

The Li‐ion battery was first introduced in 1991 and became the major energy storage device because of its high energy density (150 Wh kg^–1^).^89^ It benefits from the wide knowledge of intercalation chemistry developed by solid state chemists in the 1970s.^5^ In the first Li‐ion cell LiCoO_2_ and carbon (coke) were used as positive and negative electrodes, respectively. The electrolyte was composed of LiPF_6_ dissolved in a mixture of propylene carbonate and diethyl carbonate.^89^, ^98^ The safety of the Li‐ion battery depends mainly on thermodynamic stability of the working electrodes with respect to the electrolyte, thus the materials operating at moderate voltages are intrinsically safer.^5^, ^38^ So to keep batteries safe, graphite has remained the dominant commercial anode material for a long time, but it is not suitable as a practical anode for EVs due to its low theoretical capacity (372 mAh g^–1^).^99^ Thus, the major research in the fields of Li‐ion battery is focused on the development of new anode materials to make it applicable for EVs to preclude the production of greenhouse gases by replacing petroleum usage. Basically, the development of anode materials can be explained by the mechanism of Li^+^ storage. Initially, the storage of Li^+^ occurs through the insertion phenomenon in the existing vacancy in anode materials. However, the low capacity remains a big challenge because most of the vacancies are not available for reversible storage of Li^+^.^68^, ^100^ Later, alloying and de‐alloying of lithium with anode materials is used to store the Li^+^ and the commercially available Li‐ion battery are using same phenomenon of energy storage for portable electronics. Although an improvement in capacity resulted from the alloying mechanism, the concurrent large volume changes that led to the structural disintegration of electrode significantly compromised the performance.^5^ The third generation of anode materials works on the conversion reaction between anode materials and Li^+^, demonstrating high reversibility with improved capacity.^101^ However, a structural shrinkage due to volume expansion and voltage hysteresis is still a hurdle in their commercialization.^35^

It is well‐known that conventional graphite cannot deliver high power densities as anode of Li‐ion battery due to sluggish diffusivity of Li^+^. The emergence of graphene in 2004^102^ provided a breakthrough to resolve many issues relating to anode materials because of its high surface area, mechanical toughness, and excellent conductivity, which helped to buffer the structural changes, provide large electrode‐electrolyte contact and improve the conductivity of electrode.^103^ Graphene itself is electrochemically active towards lithium storage and has been extensively studied as anode of Li‐ion battery.^85^, ^104^ A simple photoflash and laser‐reduction of free standing graphene paper is utilized to enhance its performance as anode of Li‐ion battery as the presence of oxygen reduces the conductivity of graphene.^104^ Photothermal reduction of GO expands its structure and produce micrometer scale pores, cracks, and voids in sheets and thus open pore structure enables easy access of Li^+^ and facilitate efficient intercalation kinetics even at higher current densities. It is also observed that graphene offers nice hierarchal structures by simple growth of graphene on graphene and it further avoids the use of a binder (electrically non‐conductive in nature), thus bringing a flexible electrode structure that delivers higher performance due to its large surface area.^105^ Furthermore, it is found that heteroatom doping can improve the conductivity and electrochemical properties of graphene by breaking its electroneutrality.^106^, ^107^, ^108^ Further, heteroatom doping can significantly change the electronic structure and density of state, thus improving the interfacial energy storage and quantum capacitance of graphene.^108^, ^109^, ^110^ Keeping in mind the advantages of heteroatom doping, various electrodes have recently been developed by doping of phosphorus, nitrogen, boron, and sulfur.^89^, ^111^, ^112^, ^113^, ^114^ It is found that after doping of these elements, the electrochemical performance of the graphene has improved a lot and it brings almost double capacity than an ordinary graphite electrode.^113^, ^115^ However, it is also observed that co‐doping of two or three different heteroatoms in graphene planes alter its electronic structure in better way and enhanced its Li^+^ storage ability.^110^

**Figure 3:**
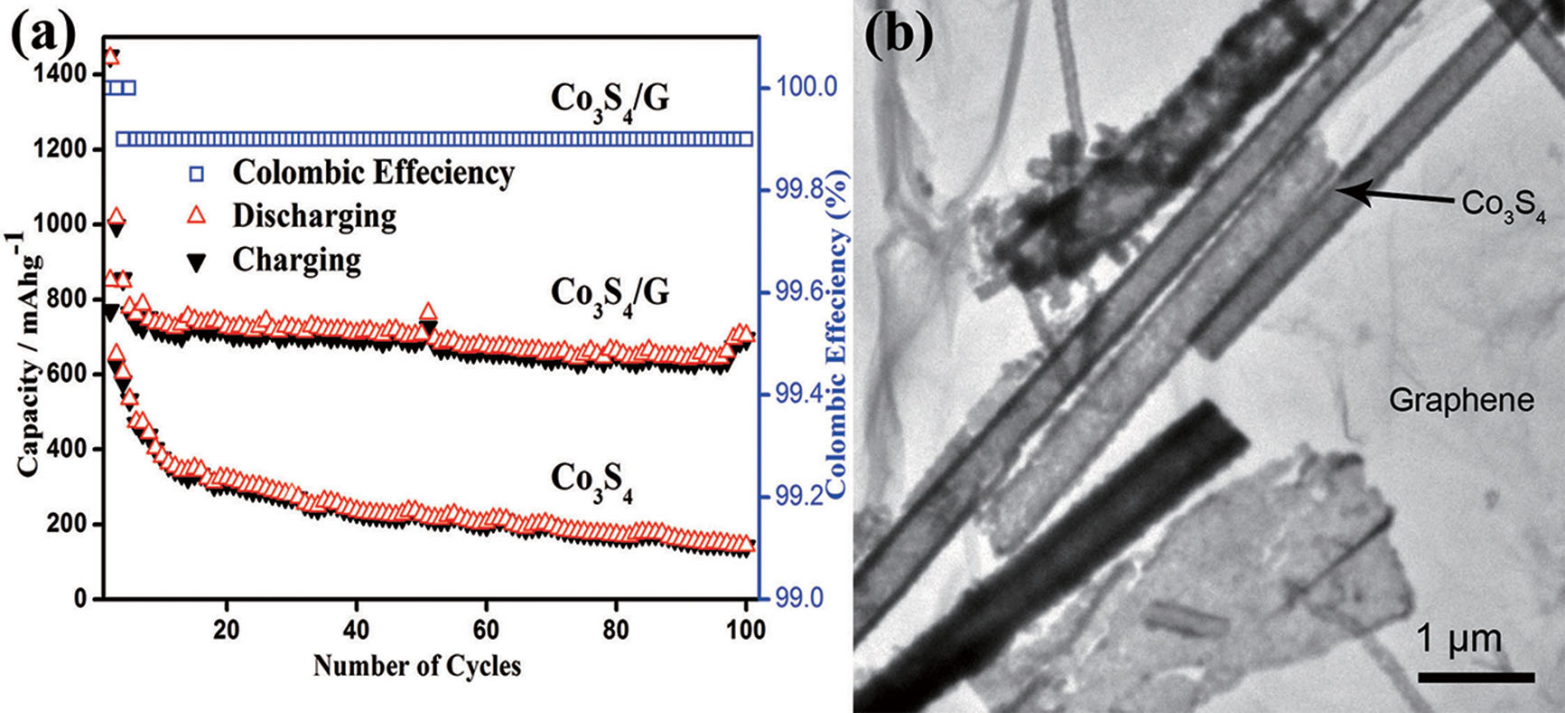
a) Cyclic behavior of Co_3_S_4_ and Co_3_S_4_/G composites with Coulombic efficiency at 0.2C rate between 0–3 V vs*.* Li^+^/Li. b) TEM image preseneting morphological aspects of Co_3_S_4_/G composites. Reproduced with permission.^120^

**Figure 4:**
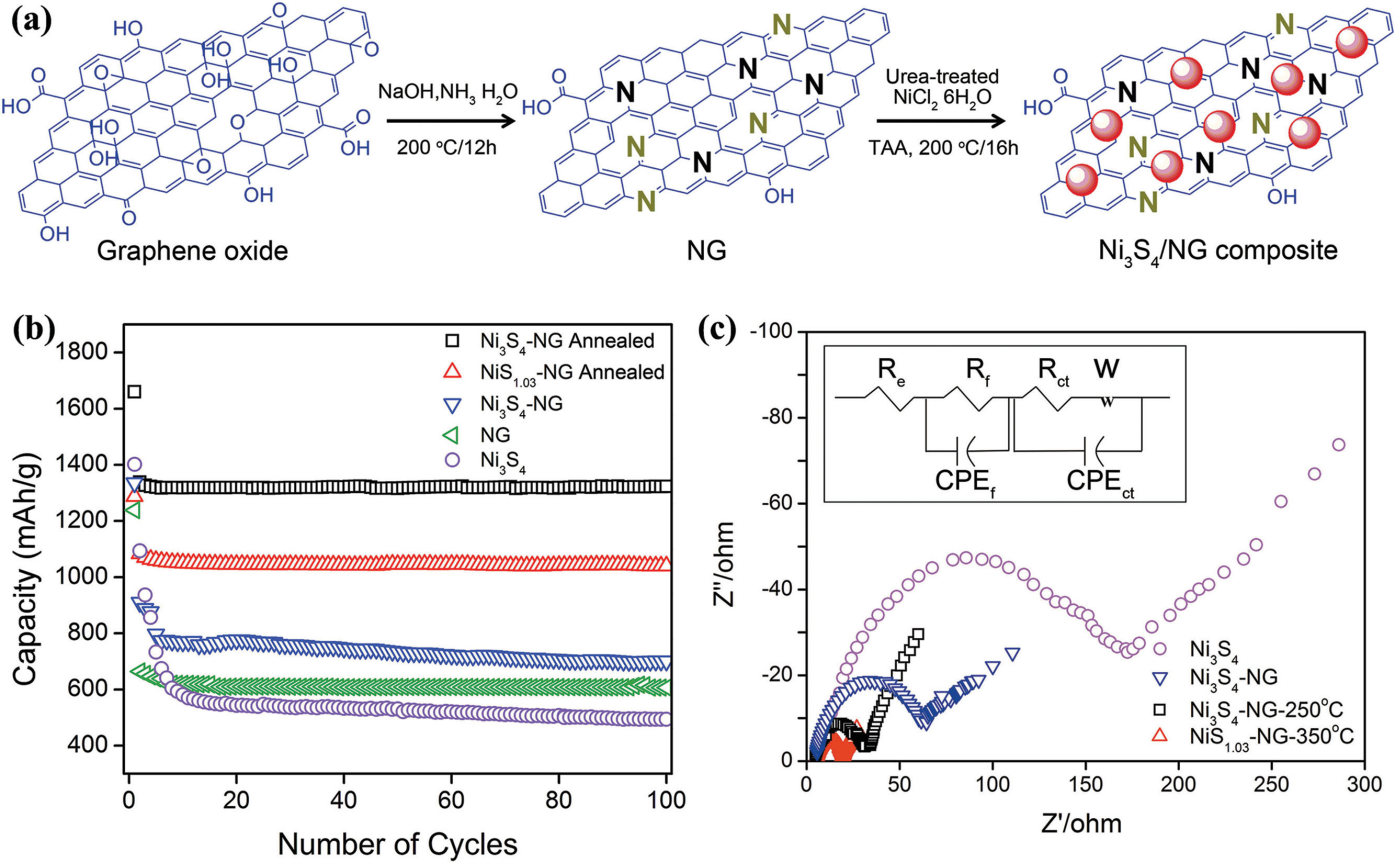
a) Schematic illustration of the preparation of Ni_3_S_4_/NG composite. b) Comparison of discharge capacities of Ni_3_S_4_, Ni_3_S_4_/NG, Ni_3_S_4_/NG‐250 °C and NiS_1.03_/NG‐350 °C at 0.2C in the range of 0‐3 V vs*.* Li/Li^+^. c) Nyquist plots for Ni_3_S_4_, Ni_3_S_4_/NG, Ni_3_S_4_/NG‐250 °C and NiS_1.03_/NG‐350 °C in the range of 100 kHz to 10 mHz. Reproduced with permission.^95^

**Figure 5:**
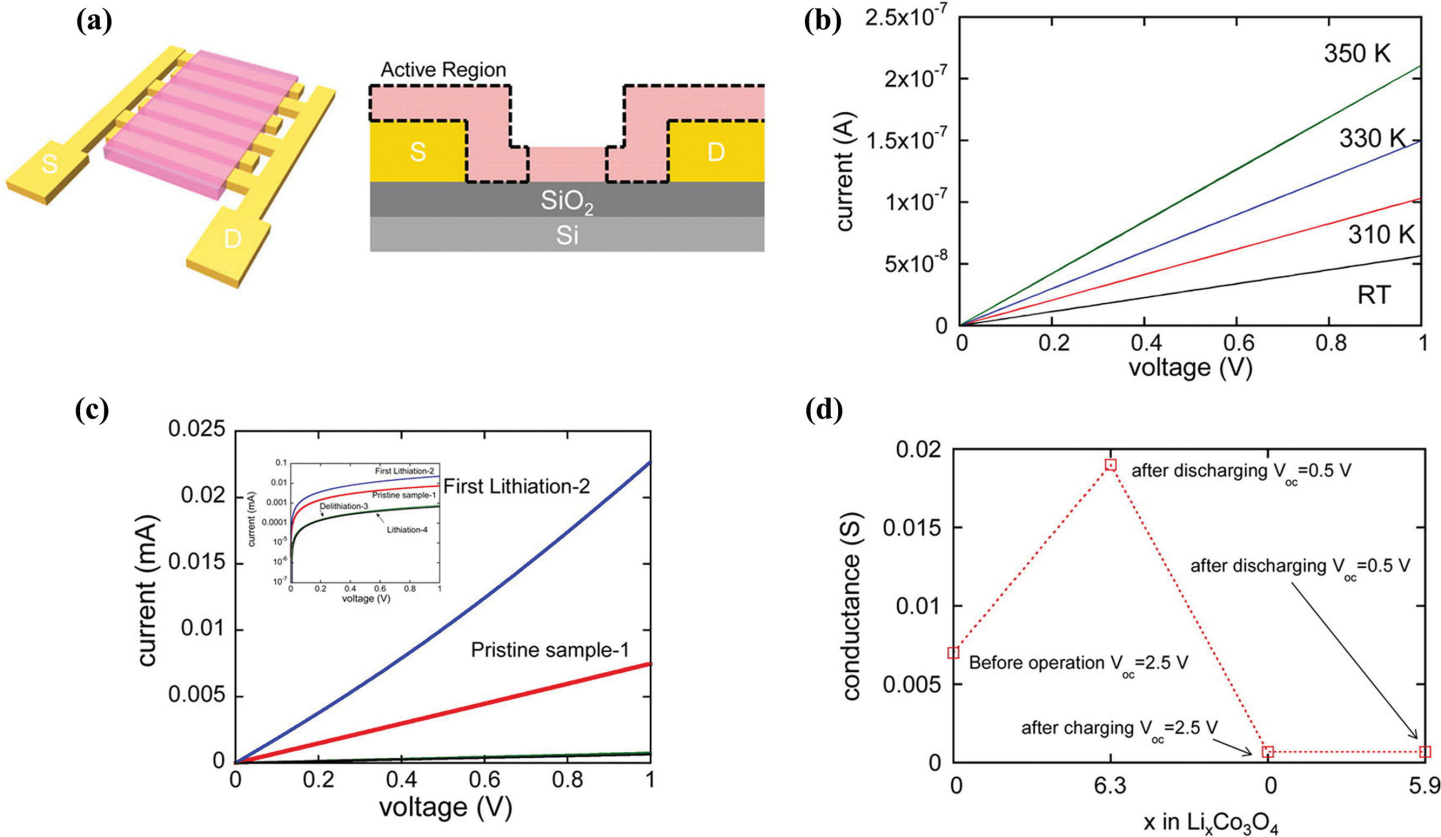
Electronic conduction measurement during charging/discharging processes. a) Schematic diagram of a two contact device with interdigitated fingers with a spacing of 20 μm. Co_3_O_4_ NPs are deposited using EPD. The active region in which Li^+^ diffuse is approximated with the dashed line. b) Temperature‐dependent I‐V curves for a thick Co3O4NPfilm. c) I‐V curves during lithiation and de‐lithiation of a Co_3_O_4_ NP film. d) Plot of conductance as a function of the Li composition change in Co_3_O_4_ NP film. The conductance value was obtained from I‐V measurements in (c). Reproduced with permission, Copyright 2014, American Chemical Society.^134^

Redox chemistry via conversion reaction provides higher energy densities, thus a rapid transition in research form intercalation to conversion occurred.^5^ In this regard, metals, their sulfides, oxides, phosphides and alloys bring very interesting performances.^25^, ^116^, ^117^, ^118^, ^119^, ^120^, ^121^ But these performances are also accompanied by new fundamental issues related to their structural change, volume expansion, uncontrolled SEI film formation, and low conductivity that results in poor cyclic life.^21^, ^37^, ^97^, ^122^, ^123^, ^124^ Among metal sulfides, Co_3_S_4_ has been considered a promising anode material for Li‐ion battery due to its high theoretical capacity (702.8 mAh g^–1^). However, capacity fading and poor cyclability because of low conductivity and production of polysulfide anions are big issues related to pure Co_3_S_4_.^125^ To overcome these problems, Co_3_S_4_ nanotubes are deposited on graphene using pre‐synthesized sacrificial nanowires (NWs) of Co(CO)_0.35_Cl_0.20_(OH)_1.10_ and C_2_H_5_NS as S source.^120^ The introduction of graphene improves the conductivity of Co_3_S_4_ while helping to preserve its structure. Furthermore, due to the porous nature of graphene, it successfully inhibits the dissolution of polysulfide anions.^126^ With good control on structure, composite brings improved cyclic life and enhanced reversible capacity compare to Co_3_S_4_ electrode as shown in **Figure** **3**a,b. A hybrid of nitrogen‐doped graphene (NG) and Ni_3_S_4_ is another example to demonstrate doped graphene nanoparticles (NPs) composite for Li‐ion battery anode, heteroatom present in graphene sheets provides anchoring sites to NPs.^95^ A higher theoretical capacity (704.5 mAh g^–1^), low cost, environmentally benign nature and safety make Ni_3_S_4_ a promising candidate for anode of Li‐ion battery. A hydrothermal reaction was carried out to sulfurize the Ni_2_(CO_3_)(OH)_2_ using C_2_H_5_NS in the presence of NG at moderate conditions (200 °C for 12 h, shown in **Figure** **4**a). The NG is synthesized by simply treating GO with ammonia solution in the presence of NaOH at 200 °C for 12 h. Here, NaOH benefits the composite with production of large size sheets for maximum loading of NP. To achieve better control over the SEI film thickness that badly effect Coulombic efficiency in the initial cycles, post thermal treatment was utilized to significantly reduce the remaining oxygen groups from surface of NG. It is well‐known that during annealing, sulfur evaporates from its compounds; thus by controlling the annealing temperature another phase of nickel sulfide was also deposited (NiS_1.08_), as confirmed by x‐ray diffraction studies (Figure 4b).The Ni_3_S_4_‐NG hybrid shows much improved reversible capacity (1323.2 vs. 494 mAh g^–1^) and capacity retention (98.9 vs. 45.2%) compared to pure Ni_3_S_4_ (Figure 4c). Furthermore, the reaction mechanism of Ni_3_S_4_‐NG and NiS_1.08_‐NG shows that Ni_3_S_4_ phase of nickel sulfide is more favorable for higher Li^+^ storage due to its ability to store larger number of Li^+^ per molecule Electrochemical impedance spectroscopy (EIS) is a good characterization tool to measure the effect of nitrogen doping, removal of oxygenated groups, different phases and to explore the synergism among various constituents of hybrid structure.^40^, ^83^, ^127^ Figure 4d,e show the EIS spectra and cyclic performance of various samples, bare sulfides, composited ones and annealed at different temperatures; from these results it can be concluded that nitrogen doping and annealing both improved the Li^+^ diffusion and conductivity of hybrid, thus improving the cyclic life and performance. Owing to its spatial 2D layered structure and good electronic properties, MoS_2_ also received tremendous attention from researchers and great advancement occurred to develop MoS_2_ anode for Li‐ion battery. The unique structures offer sandwich layers of S‐Mo‐S that are attached together by weak Van der Waals attractions with an inter‐layer spacing of 0.62–0.69 nm.^14^, ^91^ It makes MoS_2_ suitable as anode material application at a low‐potential; during its conversion reaction MoS_2_ converts to Mo and formation of Li_2_S occurred by the uptake of Li^+^.^128^ This result in significantly higher capacity compared with the intercalation chemistry of graphite‐based anode. Due to its layered structure it can easily be assembled with graphene to improve its capacitance and rate capability. It is found that l‐cysteine can easily assist the layer‐by‐layer hybrid formation of graphene and MoS_2_ and the resulted structure appear as a 3D network that brings very exciting results regarding capacitance, rate capability and improved cyclic life.^129^ Meanwhile, nanostructured transition metal oxides are also a focal point of researchers as an anode of Li‐ion battery due to their high specific capacity and excellent cycle reversibility. Importantly, their reaction mechanism with Li^+^ is similar to that of metal sulfides and is distinctly different from the classical storage of Li^+^ via intercalation and de‐intercalation process.^130^, ^131^ However, an abrupt structural change occurred during the Li^+^ reaction with metal oxides and the formation of conversion products (metal and Li_2_O) happened.^69^, ^132^, ^133^ Irreversible formation of Li_2_O causes capacity loss in the initial cycles and results in the formation of insulating layer on the surface of electrode, which results in poor capacity retention. The origins of the irreversible capacity loss in metal oxides were addressed via probing changes in the structural and electronic properties of hollow Co_3_O_4_ NPs during its conversion reaction with Li^+^ using electrochemical Co_3_O_4_ transistor devices that function as an anode of Li‐ion battery. It is found that in the first lithiation process, initially conductivity increased due to the production of metallic Co but at the same time a decrease in conductivity was found during de‐lithiation process due to the remittance of Li_2_O.^134^ It is also observed that in the later lithiation process, the conductivity remains lower as observed in the first cycle due to irreversible changes in structure during the first cycle.^135^ The observed behavior of the in situ transistor measurements is presented in the **Figure** **5**, which shows that internal changes in structure destroyed the normal diffusion path and conductivity as observed in the pristine metal oxide.^34^, ^134^

**Figure 6:**
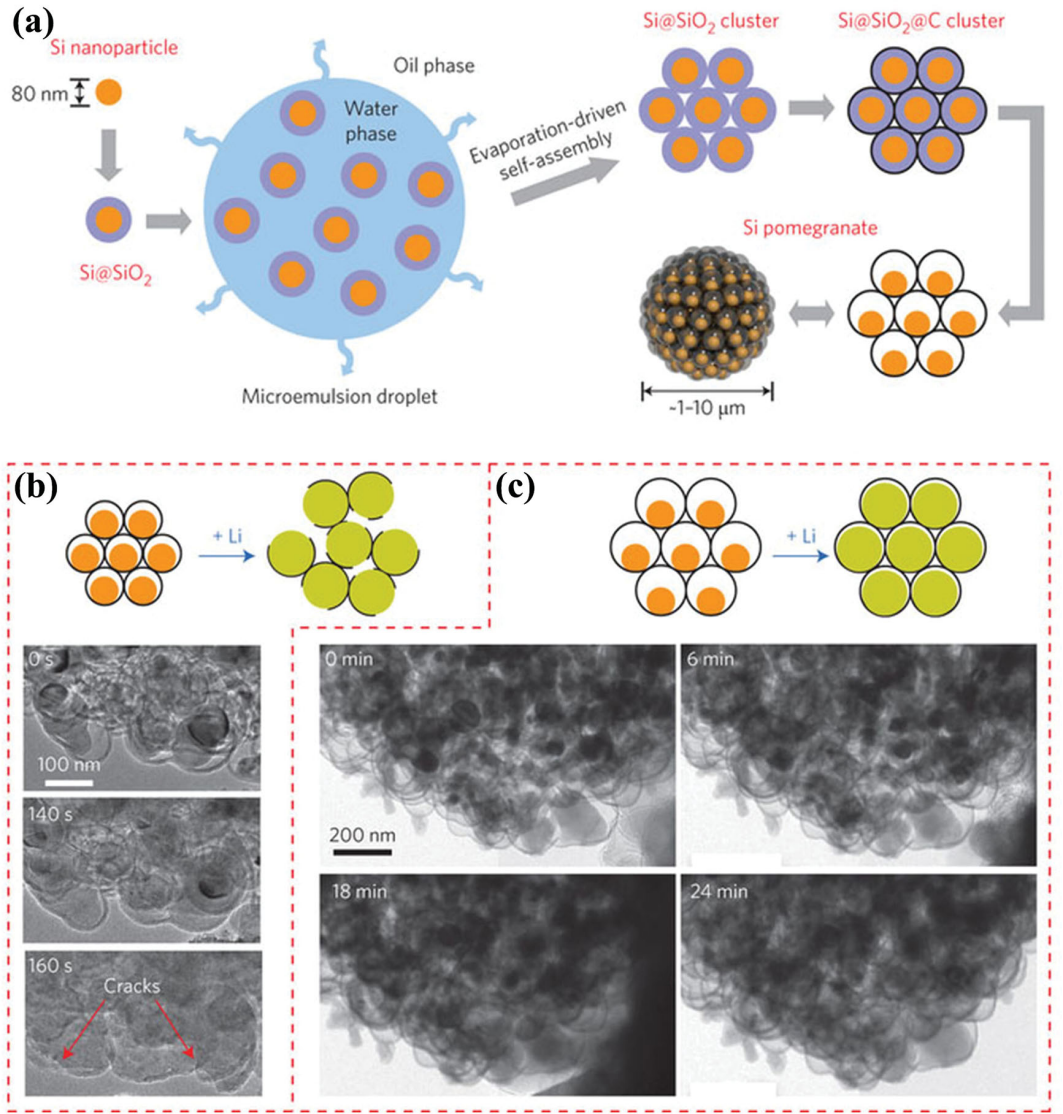
a) Schematic of the fabrication process for silicon pomegranates. b) Schematic and time‐lapse images of the lithiation of silicon pomegranates with insufficient (≈15 nm) void space. Lithium transports along and across the carbon framework to react with the silicon inside, causing volume expansion. Because the void space is insufficient, the carbon framework is ruptured by the expansion of the silicon, and the overall morphology is destroyed. c) Lithiation of a silicon pomegranate with sufficient (≈40 nm) void space; the nanoparticles expand within the carbon framework and the carbon framework does not rupture. The secondary particle morphology is therefore intact on lithiation. Reproduced with permission.^52^ Copyright 2014, Macmillan Publishers Ltd.

**Figure 7:**
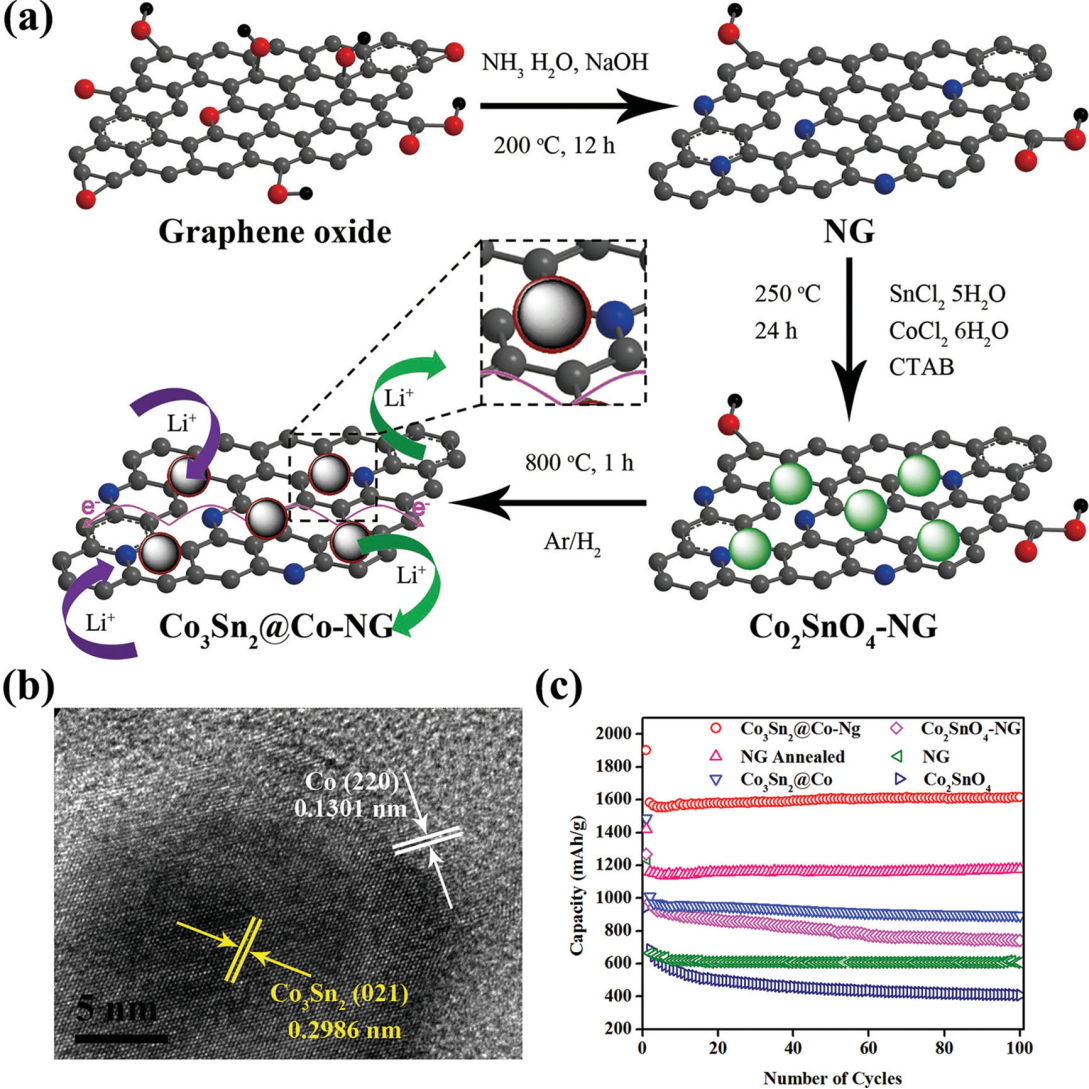
a) Schematic illustration of synthetic method of Co_3_Sn_2_@Co‐NG hybrid and its electrochemical mechanism for reversible Li^+^ storage; conductivity enhancement effect of NG. b) HRTEM image of Co_3_Sn_2_@Co‐NG hybrid. c) Comparison of discharge capacities of Co_2_SnO_4_, NG, Co_2_SnO_4_‐NG, Co_3_Sn_2_@Co, NG‐annealed and Co_3_Sn_2_@Co‐NG hybrid tested at a current density of 250 mA g^–1^ in the range of 0.005‐3 V (vs. Li^+^/Li). Reproduced with permission.^85^ Copyright 2013, American Chemical Society.

Currently, nanostructures based on group 14 (IVA) elements (Si, Ge and Sn) have given birth to a new generation of Li‐ion battery electrode materials and have shown effective improvement both in energy density and power density. Due to higher theoretical specific capacities and energy densities, these materials have become the major area of intrest for the research community to develop new electrode materials.^31^, ^41^, ^42^, ^44^ However, poor electrical conductivities and larger volume expansions (up to 300%) during lithiation and de‐lithiation process are the basic limitation of these materials.^1^, ^3^, ^12^, ^22^ The expansion and contraction during alloying and de‐alloying with Li^+^ produce stress in the electrode, resulting in structural disintegration, ultimately leading to battery failure or poor cyclic life. Several strategies have been reported including control on morphology, hybrid structures, coatings, and doping with carbon, highly conductive, hard and inactive metals and void engineered structures to accommodate the volume changes.^9^, ^33^, ^35^, ^47^, ^50^, ^124^, ^136^, ^137^, ^138^, ^139^ To overcome these issues, good control on morphology, structure and composition (homogeneity in case of hybrid structure) is required to facilitate the higher conduction of ions and electrons along with a good control on structural integrity. Recently, 0D‐in‐1D design was developed to overcome the volume changes of Ge to enhance its conductivity with faster transfer of ions.^41^ Organic‐inorganic hybrid of Ge/ethylenediamine (Ge/EDA) was developed simply using solvothermal method; Fe_3_O_4_ was used to assist the 1D growth of Ge/EDA. Further, annealing under reducing atmosphere brings unique structure (0D‐in‐ID) of NWs that are composed of carbon coated NP along with the additional sheath of carbon on NWs. This 0D‐in‐1D structures results in high capacitance with good capacity retention after long cyclic life because carbon coating around individual particles protect surface reactions and maintain faster highway for electron transfer. The void engineering is widely used to accommodate the volume changes of high capacity anode materials.^82^, ^124^, ^140^ However, only leaving the space without keeping in mind several critical issues like the extent of volume expansion this strategy is not very helpful. This is because if the void space is small and cannot accommodate the volume expansion, then it will cause the fracture of the fragile SEI film and expose the new surface to electrolyte, which results in fading of capacity.^52^ A pomegranate inspired structure was developed to control the volume changes of the silicon‐based anode of Li‐ion battery and to observe the effect of void space. This structure explains that by developing the core shell structure of individual Si NP through void engineering and then several core shell NPs are combined in the form of cluster through an additional external carbon coating to resolve the issues related to volume changes (**Figure** **6**a). Thus internal primary NPs can accommodate the volume changes within the void space and have no effect on the size of secondary particle during the lithiation and de‐lithiation process. Furthermore, the external carbon coatings act as a barrier for electrolyte and limit the SEI formation only on the surface of secondary particle and maintain the electrical conduction of electrons. Further, by utilizing in situ transmission electron microscopy (TEM), the effect of void space on the stability of structure, SEI thickness and performance was observed, and it is found that if the space is not enough for volume expansion then it causes structural disintegration (Figure 6).^52^ Further, the use of self‐healing materials is another way to control the volume changes of anode materials, like self‐healing polymer was established to control the structural damages of silicon‐based electrode. Self‐healing polymer recover the cracks or other damages in structure during conversion reaction with Li^+^.^84^ So these strategies improve the cyclic life, capacity and capacity retention of silicon electrodes. However, poor intrinsic conductivity and large volume expansion are drawbacks of several anodes, which results in limited access to redox sites and destruction of electrode.^141^ But to improve the capacitive performance, stability, and capacity retention, introduction of cobalt (internal doping and coating on Sn NP) and NG was achieved that successfully control the aforementioned problems. Initially, the NPs of Co_2_SnO_4_ was pinned on NG by hydrothermal method and the successive annealing under reducing atmosphere results in Co_3_Sn_2_@Co‐NG (**Figure** **7**a).^85^ The doping of the Co to Sn core can increase the internal conductivity, thus making accessible the redox sites and bringing structural stability because of its mechanically hard nature. Furthermore, the sealed Co coating could prevent the direct interaction of Sn with electrolyte, reduce the lithium dendrite formation that destroys the electrode structure, and control the thickness of SEI film due to its inactive nature. Furthermore, the elastically strong, flexible, and conductive NG overcoat accommodates the volume changes, therefore benefiting the structural and maintains electrical stabilization of Co_3_Sn_2_@Co NPs. In addition, the effect of oxygenated functional groups on the thickness of SEI film was also explored simply by annealing the graphene under a reducing environment. It is found that after annealing a minimal irreversible capacity loss was observed due to uniform and controlled thickness of SEI film (Figure 7b). To verify the effect of Co doping, core‐shell structure and graphene overcoat, EIS studies were utilized and the results confirmed that Co doping and coating with NG have large effect to improve the internal conductivity and diffusion of Li^+^ (Figure 7c). As a result, Co_3_Sn_2_@Co‐NG hybrid displays much improved reversible capacity of 1615 mAh g^–1^ after 100 cycles while maintaining high Coulombic efficiency of ≈100%, excellent capacity retention of 102% and good rate capability.

### 5.2 Cathode Nanostructures for Li‐ion Battery

**Figure 8:**
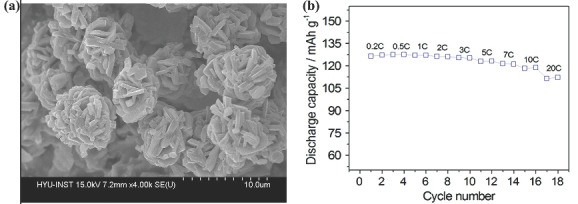
a) Field emission scanning electron microscopy (FESEM) images of the Li[Ni_0.45_Co_0.1_Mn_1.45_]O_4_ spinel electrode. b) Cycling performance of the galvanostatic test run at various C‐rates of Li[Ni_0.45_Co_0.1_Mn_1.45_]O_4_ electrode in a lithium cell (1C = 132 mAh g^–1^). Reproduced with permission.^144^ Copyright 2011, American Chemical Society.

**Figure 9:**
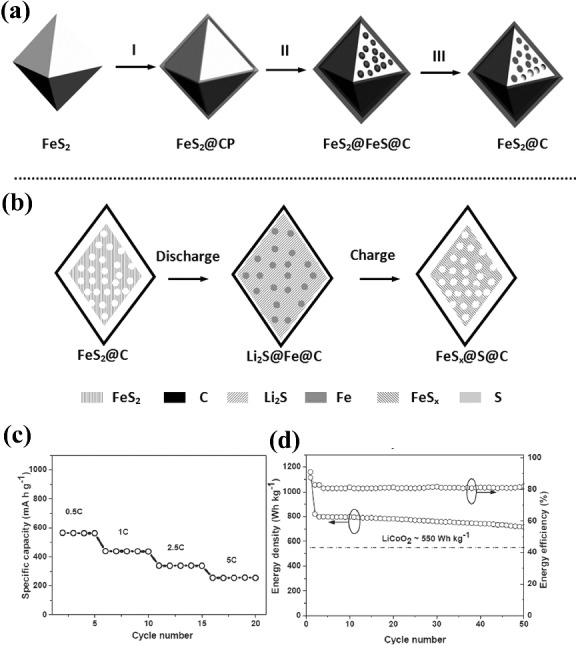
a) Schematic illustration of the fabrication of FeS_2_@C porous nanooctahedra cathode: I) uniform coating of carbon‐rich polysaccharide (CP) layers onto FeS_2_ octahedra; II) carbonization of CP layers and partdecomposition of inner encapsulated FeS_2_; III) removing of acid soluble FeS originated from the decomposed FeS_2_ by hydrochloric acid. b) Details of the discharge and charge processes of FeS_2_@C porous nanooctahedra cathode in schematic illustration. c) Rate capability at different rates (increased from 0.5C to 5C); d) discharge energy density and energy efficiency of the FeS_2_@C porous nanoctahedra vs.cycle number shown along with the theoretical discharge energy density of LiCoO_2_ cathode (550 Wh kg^–1^ based on the mass of LiCoO_2_ only), which is calculated using an average voltage of 3.9 V and a capacity of 140 mAh g^–1^. Reproduced with permission.^148^

The high energy density of Li‐ion battery makes them a promising candidate for EVs, but their low power density brings a hurdle for its application to EVs. The reason behind the low power density of Li‐ion battery is slow reaction kinetics and poor electrical conductivity of cathode materials. The commercial Li‐ion battery cathodes are composed of LiCoO_2_ that displays flat operating voltage of 3.9 V vs. Li^+^/Li among the most of lithium insertion cathode materials.^38^ Thus new cathode materials that can work with the present voltage window of the organic or solid polymeric electrolytes are highly desirable to boost up the energy and power density along with longer cyclic life of a Li‐ion battery. Several alternative transition metal oxides and phosphates are investigated as cathode, their structure was tuned with help of nanotechnology and their low conductivities are improved by making their composites with carbon, graphene and other high conductive materials.^68^, ^142^, ^143^, ^144^ By reducing the size of these materials diffusion of Li^+^ inside the electrode was enhanced by shortening the diffusion path by developing nanostructures. The insertion sites of Li^+^ are also increased by creating higher vacancies and developing nano‐morphologies of these materials or by increasing inter layer spaces.^2^ The alternative to LiCoO_2_, LiMn_2_O_4_ is also a strong candidate but display sluggish reaction kinetics and structural instability, which are big limitations towards progress. Recently by controlling the particle size to improve the reaction kinetics and doping of metals to increase the working potential, a unique cathode (Li[N_i0.45_Co_0.1_Mn_1.45_]O_4_) material was designed (**Figure** **8**a). The redox reaction was supported by the oxidation state change in doped metal and a higher working potential up to 4.7–4.8 V was attained. The resulted cathode shows excellent performance with extraordinary rate capability and retains 94% capacity as moving from 0.2 C to 10 C (Figure 8b).^144^ It is also reported that the existence of the doped metal in various oxidation states strongly affect the morphology and structure of these kind of cathode materials (Li[N_i0.45_Co_0.1_Mn_1.45_]O_4_).^145^ Furthermore, during charge‐discharge process, shift in oxidation state strongly affect the electrochemical behavior of electrode.^146^ LiFePO_4_ is also considered an optimistic electrode material with a specific theoretical capacity of 175 mAh g^–1^, but the aforementioned problems are also associated with this advanced cathode material. It was suggested that by tuning its morphology (e.g. nanorods, nanocrystals, etc.) and developing continuous thin carbon sheath to protect the structure and enhanced conductivity for faster electronic and ionic movements. Simply by changing the time and temperature, two different morpho­logies were synthesized using high temperature liquid‐phase reduction method to observe the effect of morphology on electrochemical performance of LiFePO_4_. The rhombic shape nanocrystals shows a higher performance then rod shaped one due to smaller size and higher active sites available on surface. Further, it is also concluded that carbon coating significantly enhanced the capacity and improved the rate capability.^142^ Considering the cost effectiveness and environmental issues, a waste‐to‐resource strategy was developed to convert waste bacteria to high performance cathode materials (lithium metal (Fe, Mn) phosphates, LiMPO_4_).^143^ *Escherichia coli* bacteria have been frequently utilized to remove the phosphorus contamination from wastewater. These bacteria were used as precursors for the synthesis of LiMPO_4_ by mixing them with equimolar ratios of metal and lithium ions sequentially and subsequently heating was done to obtain LiMPO_4_ nanocrystals of 20 nm. The obtained structures contain fully carbon coated particles with coating thickness of 3–6 nm that brings high ionic diffusion in the nanocrystals. The as‐synthesized nanocrystals of LiFePO_4_ deliver 140 and 75 mAh g^–1^ specific capacity at 0.1C and 10C, respectively. In contrast to layered transition LiMO and LiMPO, compounds of iron are also promising candidates as cathode of Li‐ion battery due to their higher theoretical energy density and environment friendly nature. The higher energy density of iron compounds (fluoride and sulfide) based on their conversion mechanism instead of traditional insertion mechanism of transition metal oxides and phosphates. As an example, FeF_3_ delivers large theoretical capacity of 712 mAh g^–1^ if one considers the complete conversion of FeF_3_ to LiF and metallic Fe occurring at a voltage regime of 1.5 V by 3e^−^ transfer. However, iron fluoride are large bandgap materials thus brings more difficulties to develop iron fluoride‐based cathodes for Li‐ion battery. Recently, graphene was utilized to overcome the large bandgap to speed up the redox reaction in iron fluoride.^147^ But the use of HF in the synthesis process FeF_3_ have severe concerns with environment benignity. Thus researchers explored FeS_2_ because of its higher theoretical capacity of 894 mAh g^–1^ compared to that of layered cathode materials that deliver maximum 200 mAh g^–1^.^148^ A simple solvothermal method was presented to synthesize porous FeS_2_ octahedral nanocrystal using polyvinylpyrrolidone (PVP) as solvent and FeCl_2_ as iron precursor. Sulfur was introduced in the presence of NaOH to convert FeS_2_ (**Figure** **9**a).^148^ The resulted porous FeS_2_ exhibits much higher energy density (800 Wh kg^–1^) and retention compared to the commercial LiCoO_2_ (556 Wh kg^–1^) as shown in Figure 9bc. The higher performance comes up due to porous nature and carbon coating that enhanced the conductivity and increased the electrode‐electrolyte contact area.

### 5.3 Breathing Cathode Catalyst for Li‐Air Batteries

**Figure 10:**
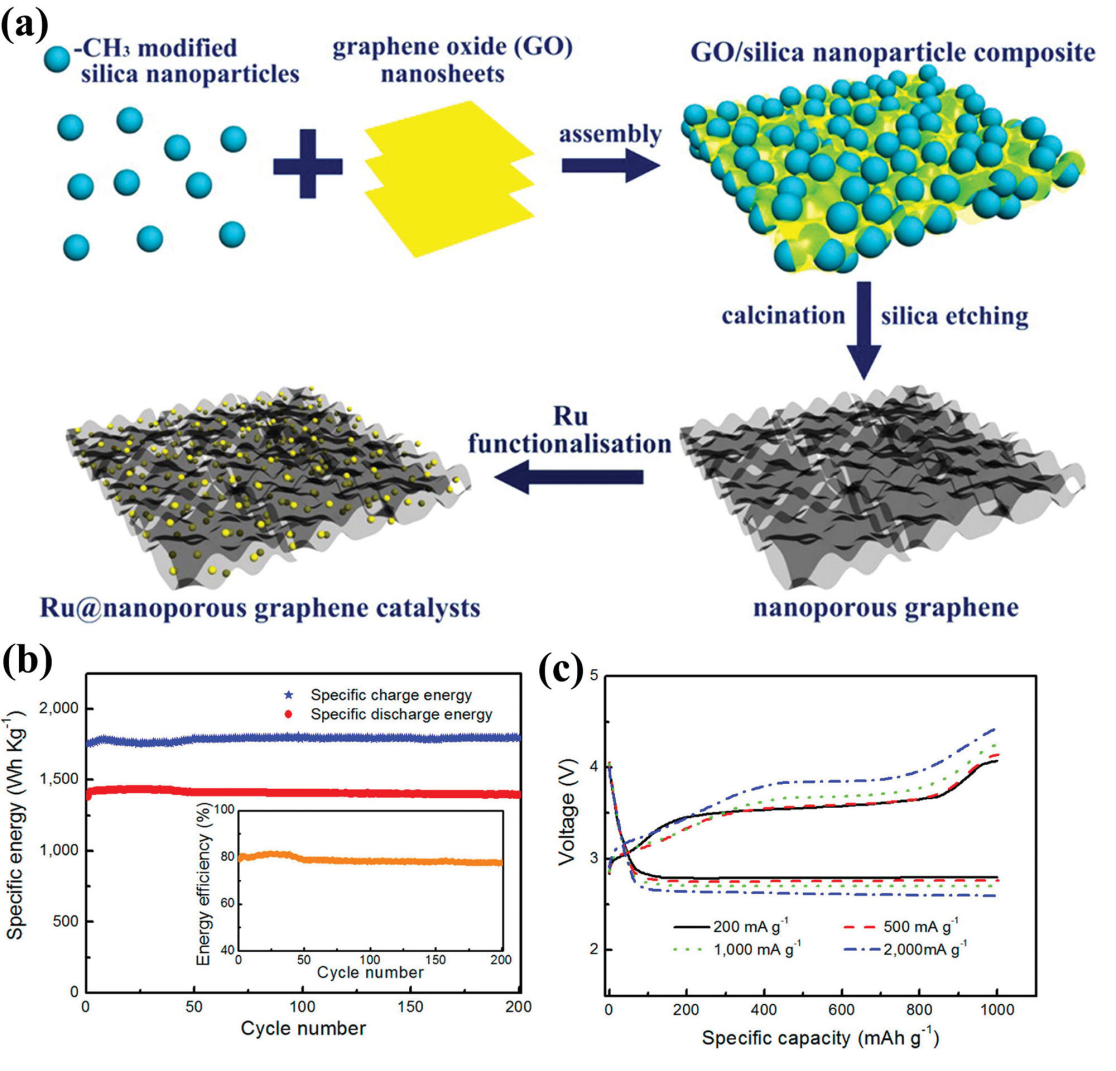
a) Schematic illustration for synthesis of porous graphene and Ru‐functionalized nanoporous graphene architectures. b) Specific energy vs. cycle number of Li‐air battery with Ru@PGE‐2 catalysts at 200 mAh g^–1^ by curtailing the capacity to 500 mAh g^–1^ in the voltage range of 2.0–4.4 V (the inset panel is the corresponding energy efficiency vs. number of cycles). c) Charge/discharge profiles of Li‐air battery with Ru@PGE‐2 catalysts at different current densities. Reproduced with permission.^87^ Copyright 2014, American Chemical Society.

The working operation of the Li‐air battery mainly depends on the structure and composition of the breathing air electrode. Because the main product of oxygen catalysis is Li_2_O_2_ that is insoluble in the electrolyte thus deposit on cathode catalyst and block the pathway of air, resulting in failure of battery operation.^149^ Thus the design of such a cathode material demands the structure which can efficiently provides the faster passage to air, excellently reduces oxygen during discharging and evolves it during charging to keep the air pathway clear for further air intake.^27^ However, the best catalyst for oxygen reduction reaction (ORR) and oxygen evolution reaction (OER) are noble metal catalysts, but their high cost is not feasible for their use in commercial Li‐air batteries.^149^ Therefore, various metal free and non‐precious metal catalysts have been explored to quantify the challenges of breathing electrode.^62^, ^74^, ^87^, ^150^, ^151^ Among the carbon‐based materials, porous graphene is a very promising candidate for the cathode of Li‐air battery as its porosity can easily be tuned and it has the ability of ORR. To observe the effect of porosity on the performance and life of Li‐air battery, graphene were synthesized with different pore sizes ranging from 60–400 nm. It is found that the graphene with pore size of 250 nm showed the highest discharge capacity than smaller and larger pores because of its higher catalytic power towards ORR and larger space to accommodate the discharge products. But continuous accumulation of discharge products block the air pathway and battery could not continue its operation after 20 cycles. Thus, OER active catalyst is highly desired to reverse the battery process for long cyclic life. The Ru NPs were decorated on porous graphene (Ru@PG) as shown in **Figure** **10**a, Ru@PG catalyst is highly active for both ORR and OER to keep the air pathway clear for longer battery operation (Figure 10b,c). As a result, the Ru@PG catalyst shows higher capacity (17 700 mAh g^–1^), low over potential increment (≈0.355 V), long cyclic life (200 cycles) and high energy efficiency (86.6%).^87^ Cobalt and manganese containing carbon is also good candidate for cathode catalyst of Li‐air battery.^65^, ^152^, ^153^ The catalytic properties of Co towards ORR and OER were observed by dispersing the fine NPs of Co on carbon substrate. It is found that after the completion of the discharge process the composite surface contains aggregation of small particles (lithium oxide) but after discharge no aggregation or nano lithium oxide was found on composite surface that confirm the high efficiency of the Co@C composite.^65^ In contrast to the high conductivity, carbon substrates easily get corroded in higher oxidative potential during the electrochemical water oxidation.^154^ Thus, highly conductive alternate catalyst substrates for breathing cathode of Li‐air battery are required. Several choices are listed by various scientists such as titanium oxide, boron carbide, nickel foam and titanium metal sheets as catalyst support as well as gas diffusion layers. Recently, stainless steel (SS) is also considered an alternative catalyst support to carbon because of its low‐cost and higher oxidation resistant. Additionally it contains traces of nickel which is also a known catalyst for OER. To evaluate the feasibility of the SS as catalyst support, manganese oxide was supported on SS and utilized as cathode catalyst for Li‐air battery. By comparing the OER performance of the hybrid structure and bare SS, it is proved that OER phenomenon majorly occurred at the surface of catalyst support (SS). To further confirm the OER active nature of the SS substrate, different mass loading of catalyst was deposited on the surface of SS and it was found that at higher mass loading OER performance of the catalyst was low. The reason behind the lower OER performance at higher mass loadings are due to less free surface of the SS remains available for OER.^149^ Thus, these results confirm that SS is very promising substrate for catalyst to use in Li‐air cell similar to carbon (as carbon is ORR active) but can bear more oxidative conditions. To avoid side reaction of carbon‐based catalyst and bring higher synergistic effect for improved ORR and OER reactions for highly stable Li‐air cell performance, catalyst based on Ru supported on Sb‐doped tin oxide (Ru@STO) was developed. The Ru@STO catalyst can recycle the battery reversibly without the deposition of reaction products as low over‐potential for charge (0.20 V) and discharge (0.35 V) is observed. The large over‐potential is mostly happened due to the accumulation of discharge products or poor stability of the electrolyte. Since, it is a well‐known fact that to achieve the stable cyclic life more than 100 cycles, electrolytes should be stable in the working potential window of lithium metal and cathode catalyst. Thus Ru@STO can catalyze the cathode reaction below 4.0 V where electrolyte is stable and brings higher capacity of 750 mAh g^–1^ with longer cyclic life of 50 cycles.^154^ This higher performance of the hybrid is attributed to the high conductivity of substrate and stronger interfacial interactions. In short, to bring the stability and keep higher performance for longer cycles, it is necessary to design well controlled cathode catalyst that can avoid side reaction, maintain continous breathing of cell and work in the stable potential window of electrolytes.

### 5.4 Sulfur Electrode for Li‐S Batteries

Currently a particular attention is devoted to super batteries that have the energy density up to several hundred watt‐hour per kilogram, which are in fact regarded as the sole electrochemical systems. Among these systems, Li‐S battery is at advanced state of development due to a series of recent breakthroughs that overcame several operational issues. The high theoretical capacity (1672 mAh g^–1^), low price, natural abundance and environmentally benignity make sulfur a promising material to be used as electrode in Li‐S battery.^89^ The Li‐S battery possess a high theoretical specific and volumetric energy densities of 2600 Wh kg^–1^ and 2800 Wh L^–1^, respectively by considering the complete formation of Li_2_S that may be successfully applied to electrify the road transportation.^67^ But several challenges still need to be addressed properly before the market availability of these batteries. Here we will briefly describe these issues with possible solutions and efforts made to solve them. The first and the most important problem is the formation of the polysulfide anions (byproducts of sulfur) that can travel from one electrode to another and react there to produce lower order polysulfide anions. These lower order polysulfide anions could travel back due to concentration gradient called polysulfide shuttle that badly affect the performance by disturbing the transportation of Li^+^.^89^ Furthermore, these polysulfide anions are easily dissolved in liquid organic electrolytes and the dissolution of these polysulfide anions lower the conductivity and destroy the structure of cathode by reacting at surface of electrode (dendrite formation).^56^ Several solutions have been proposed to this problem such as: 1) use of solid electrolytes like liquid free polymer electrolytes.^56^, ^155^, ^156^ In addition, use of electrolytes additives that limit the dissolution of polysulfide anions in the electrolytes such as solvent‐salt complex [acetonitrile(ACN)_2_‐LiTFSI] with a hydrofluoroether co‐solvent. The resulted Li‐S battery delivered 1000 mAh g^–1^ capacity with excellent capacity retention up to 100 cycles.^60^ 2) The synthesis of sulfur hybrid structures with porous carbon or/and graphene that can adsorb polysulfide anions and prevent there dissolution in electrolyte.^67^, ^157^ 3) The use of selectively permeable separator is another possible way to overcome the polysulfide anion shuttle that has ability to block polysulfide anions and provide free passage to Li^+^.^158^ 4) By developing nano‐morphologies of sulfur alone or encapsulating it into a mesoporous carbon matrix, or preparing sulfur impregnated graphene paper and graphene wrapped sulfur NPs. Additionally, developing polysulfide anions reservoirs via embedding carbon and sulfur composites in porous silica substrate is an alternative way.^67^, ^159^, ^160^

**Figure 11:**
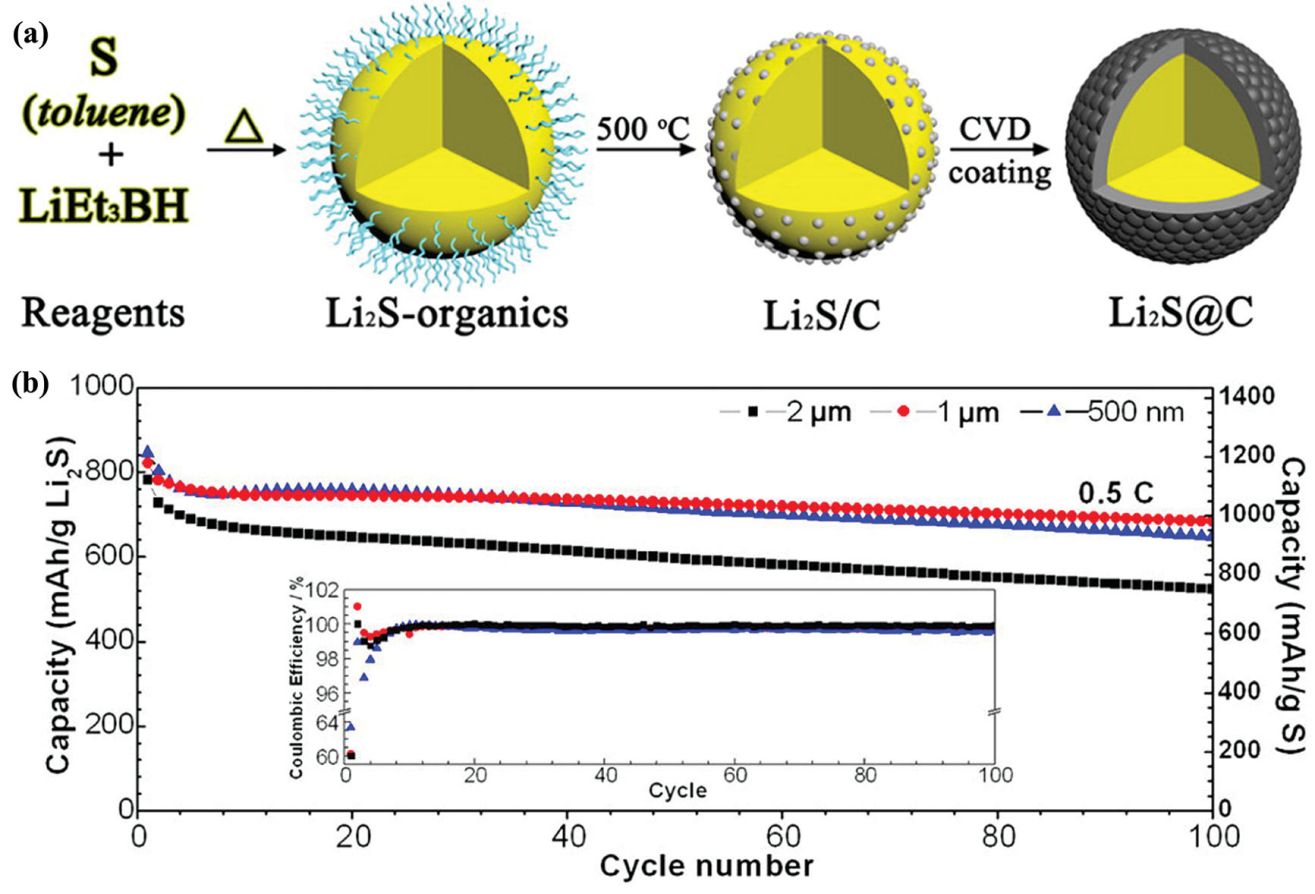
a) Schematic of the synthesis and coating process for the Li_2_S@C spheres. b) Cycling performances of the 2 μm, 1 μm, and 500 nm Li_2_S@C particles at the 0.5C rate (the inset is corresponding Coulombic efficiency). Reproduced with permission.^75^ Copyright 2014, American Chemical Society.

**Figure 12:**
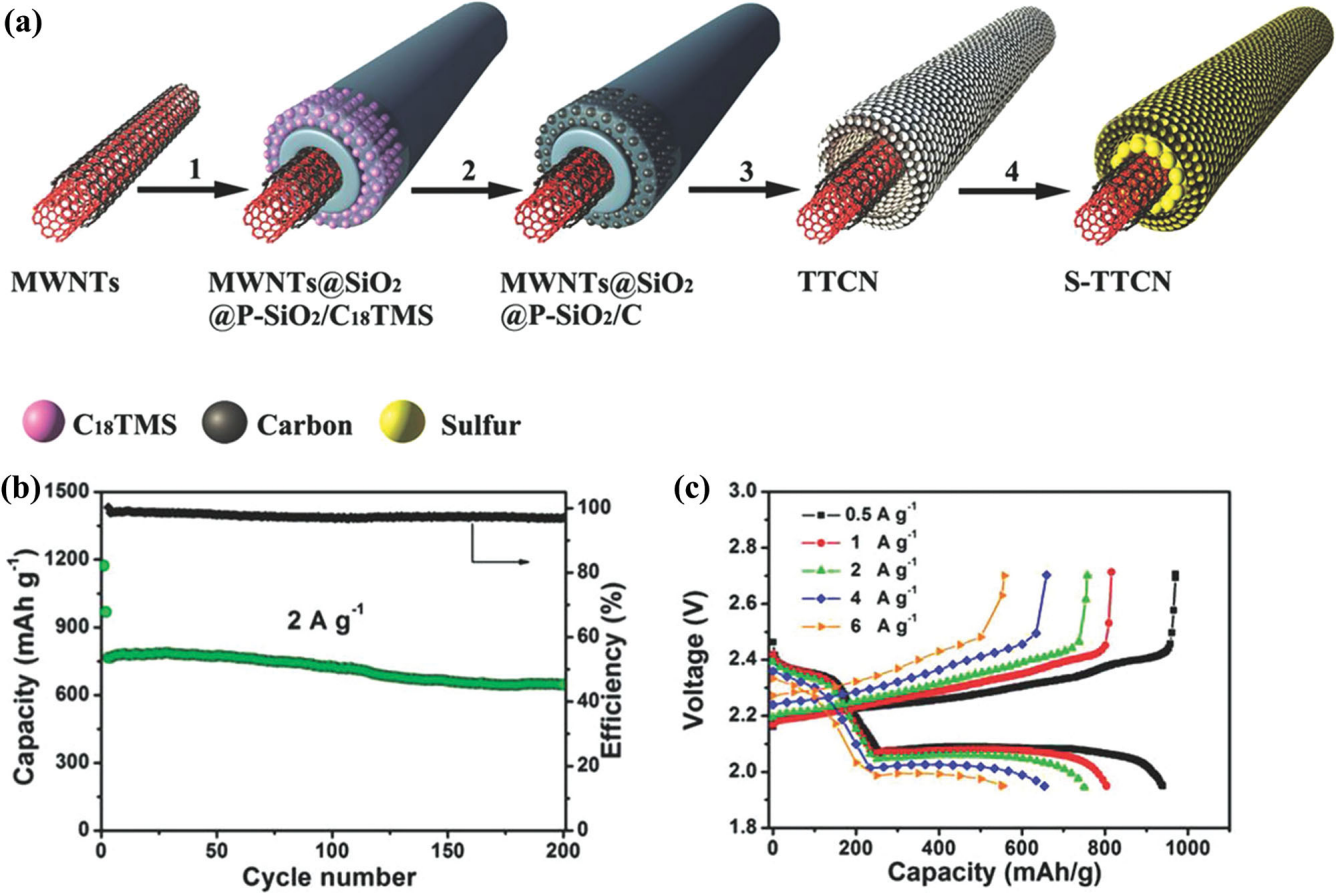
a) Schematic illustration for the formation of S‐TTCN composite: 1) Uniform coating a solid SiO_2_ layer and a porous SiO_2_ layer embedded with C_18_TMS molecules on MWNTs; 2) formation of porous carbon nanotube by carbonization of C_18_TMS; 3) etching SiO_2_ layers to obtain tube‐in‐tube carbon nanostructure (TTCN) with MWNTs encapsulated within hollow porous carbon nanotube; 4) sulfur infused into TTCN to fabricate S‐TTCN composite. b) Long‐term cycling performance of S‐TTCN electrode at a high rate of 2 A g^–1^. c) Discharge‐charge profiles of S‐TTCN at various current densities from 0.5 A g^–1^ to 6 A g^–1^. Reproduced with permission.^163^

Furthermore, lower conductivity of sulfur (5 × 10^−30^ S cm^–1^) causes the poor transfer capability of ions and electrons of sulfur electrode leading to low capacity and poor capacity retention.^78^ Additionally, sulfur bears large volume expansion (76%) during charging‐discharging process that cause structural degradation and break down of specific morphology which result in fading of capacity.^161^ These problems can be solved by incorporating conductive substrate and elastic buffer like graphene or combining them with other organic or inorganic elements.^158^, ^159^ Several groups devoted their efforts in solving the aforementioned problems and enhancing the performance of the Li‐S battery by using graphene, carbon and polymeric additives.^155^, ^157^, ^161^, ^162^ The structure, size and morphology both have strong influence on the electrochemical performance of electrode materials. To address the fact of structural change and size of Li_2_S, different size particles (0.5, 1, and 2 μm) of Li_2_S were synthesized by chemical route and then coated with carbon through chemical vapor deposition (CVD) as shown in **Figure** **11**a.^75^ It is concluded that carbon coating successfully overcame the aforementioned problems and electrochemical results show highest performance for 1 μm particles (Figure 11b). To enhance the conductivity and control the structural changes of sulfur electrode, sulfur particles incorporated graphene paper is fabricated.^162^ The graphene paper serves both as conductive network and elastic buffer to relax the strain caused by volume change of sulfur and bring higher energy density (804 Wh kg^–1^). However, the open ends of graphene cannot control movement of polysulfide anions across the cell and their dissolution in electrolyte. One possible solution to overcome this issue is to use solid electrolyte to further improve the performance by solving the problem of polysulfide anions dissolution but the slow transfer rate of ions remains a point to ponder. Furthermore, the synthesis of graphene sheets with dense nanopores on surface of graphene through chemical activation of rGO *via* hydrothermal process were achieved.^160^ A uniform confinement of the sulfur into the nanopores of activated graphene sheets were attained, where sulfur has intimate electrical contact with the graphene framework. As the sulfur was constrained in the nanopores, the nanopores are serving as “micro‐reactors” for the electrochemical reactions, thus controlled the diffusion of polysulfide anions and relax the strain, resulting in high performance (1379 mAh g^–1^) and good cyclic stability. Another rational strategy to overcome the problem of low conductivity, volume expansion and dissolution of polysulfide anions is by developing sulfur‐graphene stacked‐up hybrid structure that is further covered by rGO to stop the leakage of polysulfide anions.^67^ The higher surface area of graphene is achieved by thermal exfoliation and sulfur was incorporated in the pores of graphene by sublimation at 150 °C, in next step rGO was coated chemically on the graphene‐sulfur composite. The resulted structure displays 100% Coulombic efficiency that confirms the high reversibility of the sulfur electrode. The Coulombic efficiency is the indication of the reversibility of the electrochemical reaction, the decrease in the Coulombic efficiency in the later cycles mostly happen due to structural or electronic changes in the electrode materials that delineate their poor capacity retention. Tube‐in‐tube strategy was developed to encapsulate sulfur, multi‐walled carbon nanotubes (MWCNTs) were acid treated and then solid and porous SiO_2_ were deposited by a modified Stöber method. To develop porous carbon tube on it, octadecyltrimethoxysilane was introduced as carbon source and then annealing was done. After etching the SiO_2_ by NaOH, sulfur was introduced simply by melt infiltration method (**Figure** **12**a).^163^ The tube‐in‐tube design maintained the electrical highway and confined the polysulfide anions inside the tubes and delivered high capacity of 647 mAh g^–1^ after 200 cycles at 2 A g^–1^ (Figure 12b,c). Recently, sulfur‐inorganic (Ti_n_O_2n‐1_) hybrid structures were also reported to resolve the concerns related to stability of Li‐S battery, the smaller band‐gap of titanium dioxide can effectively deliver the electrons to sulfur if the sulfur has strong electrochemical coupling with titanium dioxide and shows excellent capacity of 1342 mAh g^–1^ with capacity retention of 99%.^164^

### 5.5 Concerns with Lithium Metal Anodes

**Figure 13:**
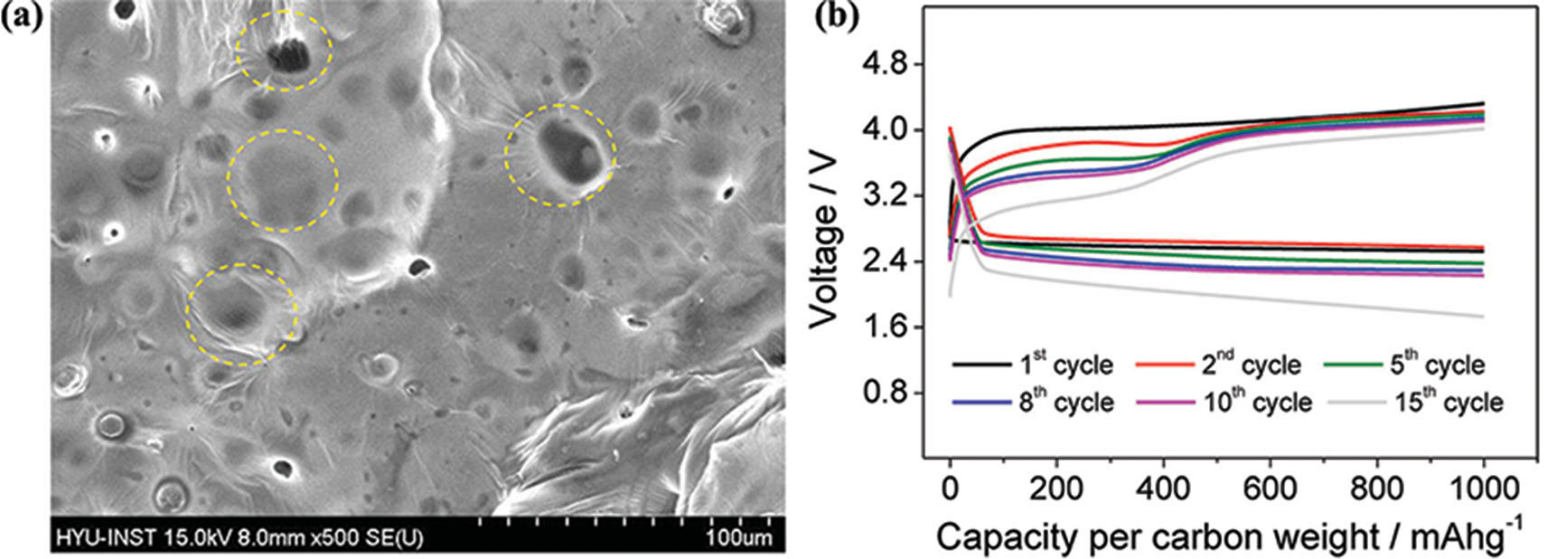
a) Scanning electron microscopy (SEM) image of the Li_x_Si electrode formed by microsized carbon particles containing nanosized lithiated silicon particles, here highlighted by yellow circles. Also the coverage by an SEI film during the lithiation process is clearly shown. b) Voltage profiles of the lithiated‐silicon/carbon‐oxygen cell. Cycling current is 200 mA g^–1^. Reproduced with permission.^151^ Copyright 2012, American Chemical Society.

The lithium metal with highest theoretical specific capacity (3860 mAh g^–1^), lowest negative redox potential of –3.040 V vs. SHE (standard hydrogen electrode) and lower gravimetric density (0.59 g cm^–3^) make it a “holy grail” for energy storage systems.^165^ Thus, lithium metal is a most promising anode material especially for Li‐air and Li‐S batteries.^166^ But the formation of the lithium dendrites due to inhomogeneous current energy distribution on lithium anode (the higher deposition of the lithium will occur at higher current density points) and irregular concentration gradient of Li^+^ at the contact surface electrode/electrolyte due to formation of LiOH are the major issues in the application of lithium metal anode for these high energy density devices. Furthermore, these dendrites have higher reactivity and produce the dead lithium from their interface that detach from electrode and does not contribute to capacity.^167^, ^168^ These dendrites can damage the polymeric separator causing short circuit of battery and as a result thermal runway of the cell. Furthermore, these lithium dendrites result in non‐uniform SEI film, thus exposed new metal surface to electrolyte and result in the re‐formation of the SEI.^86^ As the reformation of the SEI film is irreversible process thus continuously consume Li^+^ from electrolyte. In addition, the SEI film is non‐conductive to electrons thus resistances become larger with the formation of dendrite and increase the diffusion path for Li^+^ thus slowers the reaction kinetics.^89^ The morphology, composition and stability of these dendrites determine the performance of the lithium metal containing cell. If the stable interface can be achieved by developing some coating, the addition of different additive (e.g. LiPF_6_, LiBF_4_ and HF etc.) to electrolyte, use of elastically strong polymeric electrolytes, nanostructures of lithium containing compounds then the formation of dendrites can control.^76^, ^151^, ^169^, ^170^ The addition of additives like salts of fluorides can overcome the formation of the dendrites by making a stronger interface film and protect metal‐electrolyte interface. Nowadays Cs and Rb also utilize to develop a protecting coating due to their lower reduction potential than standard Li^+^/Li, but these kind of coating introduce fundamental issues as reducing lithium diffusion, increase resistances to electrons and also hinders the electrode‐electrolyte redox reaction.^76^ Instead of these in‐situ coatings, ex‐situ polymeric coatings were also employed to retrain the growth of dendrites on the surface of lithium metal. However, the results are not well‐improved, thus few groups report the utilization of the lithiated compounds of carbon or other inorganic elements to stabilize the anode of Li‐air and Li‐S batteries. Lithium containing 3D nanostructure of fibrous Li_7_B_6_ have also been developed to overcome the volume changes and formation of dendrites.^76^ The 3D architecture sufficiently provides the space to accommodate the deposition of LiO_2_ and current energy distribution to recover the metallic lithium for long cyclic life with excellent capacity retention. **Figure** **13**a is presenting the morphology of alternative anode, consisted of lithiated silicon particles embedded in carbon matrix that can successfully overcame the formation of dendrites and thickness of the SEI film.^151^ The resulted electrode shows excellent stability when tested as counter electrode along with air electrode in Li‐air battery (Figure 13b). But this kind of electrodes sufferes from particle seggration during lithiatioion/de‐lithiation process and make the reaction kinetices complicated. Another, critical problem conversion of lithium to LiOH by reacting with hydroxayl ions produced by the decomposition of electrolyte at carbon catalyst, no solution and deep investigations were yet reported about this key isue of lithium metal anode. Thus, further research is required to improve the stability concerns of lithium metal anode especially to fullfil the dream of electrification of road market by utilizing Li‐air and Li‐S batteries.

## 6 Conclusions and Perspectives

Thanks to the high energy densities, long cyclic life, safety, and enviromental beniginity of lithium‐based batteries, the dream of a continuous supply of energy to portable electronices and short‐distance traveling EVs is coming true. A series of research has been done to improve the energy storage mechanisim of Li‐ion battery by introducing new nanostructures and their hybrids with graphene/carbon that can overcome several issues, such as structural changes, low conductivity and poor capacity, stability concerns of Li‐air battery by developing new breathing catalyst having the ability of ORR and OER to keep the air pathway clear and limited electronic flow caused by the deposition of reaction products. Further, development of electrolytes and several additives is also helpful to improve the cyclic life of the Li‐air battery. Moreover, polysulfide shuttles are also controlled by developing new hybrid structures of sulfur that limits the dissolution of polysulfide anions. The concerns using lithium metal anode are also resolved by introducing new lithiated anodes for Li‐air and Li‐S batteries. However, to achieve the standards set by USABC, new synthesis methods should be developed for electrode nanostructures that can be industrially acceptable. Definition of new stable designs and/or hybrids of nanostructures of electrodes is needed that can overcome the problems of large volume changes, electrical insulation, formation of homogenous SEI film and meanwhile brings higher packing density along with access to all redox sites for larger volumatric density. Porous breathing cathodes with larger surface area bi‐functional catalysts that can maintain higher electrical conductivity and successfully reduce and oxidize oxygen with ongoing discharge‐charge process. Sulfur containing closed structures should be developed for Li‐S battery that act as micro‐electrochemical reactors, inhibiting the dissolution of sulfur while control its volume change and enanhced its conductivity. Protection of lithium metal anode from side‐reactions to form surface dendrites and conversion to LiOH by reacting with hydroxal ions produced from decomposition of electrolyte at catalyst surface. Future work on lithium‐based batteries should explore the fundamental characterizations of reaction products, the battery chemistry and the stability concerns of electrodes and electrolytes inside the battery with ongoing charge‐discharge process.
